# STAT3/miR-130b-3p/MBNL1 feedback loop regulated by mTORC1 signaling promotes angiogenesis and tumor growth

**DOI:** 10.1186/s13046-022-02513-z

**Published:** 2022-10-11

**Authors:** Hongwu Li, Ping Liu, Dapeng Li, Zixi Wang, Zhao Ding, Meng Zhou, Xu Chen, Manli Miao, Junli Ding, Wei Lin, Yehai Liu, Xiaojun Zha

**Affiliations:** 1 Department of Otorhinolaryngology, Head & Neck Surgery, The First Affiliated Hospital of Anhui Medical University, Hefei, 230032 China; 2 Anhui Public Health Clinical Center, Hefei, 230032 China; 3 Department of Biochemistry & Molecular Biology, School of Basic Medicine, Anhui Medical University, Hefei, 230032 China; 4 Department of Pharmacy, Genertec Universal Medical Maanshan Shiqiye Hospital, Maanshan, 243000 Anhui Province China; 5 Department of Pediatrics, The First Affiliated Hospital of Anhui Medical University, Hefei, 230032 China; 6 Department of Stomatology, The First Affiliated Hospital of Anhui Medical University, Hefei, 230032 China

**Keywords:** mTOR, miR-130b-3p, STAT3, MBNL1, Angiogenesis, Tumor growth

## Abstract

**Background:**

Aberrantly activated mammalian target of rapamycin complex 1 (mTORC1) plays a vital role in tumor angiogenesis, but its precise mechanisms are still unclear.

**Methods:**

Micro-RNA-130b-3p (miR-130b-3p) expression in mTORC1-activated and control cells was examined by quantitative real-time PCR (qRT-PCR). MiR-130b-3p levels and their correlation with mTORC1 activity were evaluated by analyzing publicly available databases and in-house head and neck squamous cell carcinoma (HNSCC) tissues. The role of miR-130b-3p in mTORC1-mediated angiogenesis and tumor growth was examined using tube formation assay, chicken chorioallantoic membrane assay, cell line − derived xenograft models, and an HNSCC patient-derived xenograft (PDX) model. The regulatory mechanisms among signal transducer and activator of transcription 3 (STAT3), miR-130b-3p, and muscleblind-like protein 1 (MBNL1) were investigated via bioinformatics analyses, qRT-PCR, western blot, RNA immunoprecipitation, immunofluorescence, luciferase reporter assay, and chromatin immunoprecipitation assay.

**Results:**

Elevated miR-130b-3p enhanced the angiogenic and tumorigenic abilities of mTORC1-activated cells both in vitro and in vivo. STAT3, a downstream effector of mTORC1, transactivated miR-130b-3p by direct binding promoter of the miR-130b gene. MBNL1 was identified as a direct target of miR-130b-3p. MBNL1 depletion rescued the compromised angiogenesis and tumor growth caused by miR-130b-3p inhibition. MiR-130b-3p levels were significantly upregulated and positively correlated with mTORC1 signaling in multiple cancers. MiR-130b-3p inhibition attenuated tumor angiogenesis and growth in an HNSCC PDX model. MBNL1 feedback inhibited STAT3 activation in mTORC1-activated cells.

**Conclusions:**

The STAT3/miR-130b-3p/MBNL1 feedback loop plays a vital role in mTORC1-mediated angiogenesis and tumor progression. This pathway could be targeted for therapeutic intervention of mTORC1-related cancers.

**Supplementary Information:**

The online version contains supplementary material available at 10.1186/s13046-022-02513-z.

## Background

Angiogenesis is recognized as one of the hallmarks of cancer [1–3]. Tumor-associated angiogenesis has a critical role in the delivery of oxygen and nutrients to expanding tumors, thus significantly influencing various aspects of cancer development, such as tumor cell metastasis and metabolic dysregulation [4, 5]. Because of its fundamental role in tumor progression, angiogenesis has become an attractive target in cancer treatment [6, 7]. However, the mechanisms and pathways that drive tumor angiogenesis are not fully understood.

The mammalian target of rapamycin (mTOR), a highly conserved serine/threonine protein kinase, regulates a wide repertoire of biological processes important for cell growth and proliferation, including protein translation, metabolism, autophagy, and ferroptosis [8, 9]. mTOR can form two functional complexes, mTOR complex 1 (mTORC1) and mTOR complex 2 (mTORC2), which are different with regard to specific binding components, upstream and downstream signaling, and rapamycin sensitivity [10]. The heterodimer tuberous sclerosis 1 (TSC1)/tuberous sclerosis 2 (TSC2) is an important upstream negative regulator of mTORC1 [11]. TSC1/TSC2 complex suppresses a small GTPase, Ras homolog enriched in the brain (Rheb), by stimulating the conversion of active Rheb-GTP into inactive Rheb-GDP through the GTPase activating (GAP) activity of TSC2 [12]. The activity of the TSC1/TSC2 complex relies on heterodimer formation and is controlled by several kinases, particularly AKT [13]. Disruption of the TSC1/TSC2 complex by the activated PI3K/AKT pathway, or by inactivating mutations in either TSC1 or TSC2, results in the accumulation of GTP-bound Rheb, which in turn activates mTORC1 [14]. mTORC1 promotes protein synthesis through phosphorylating ribosomal protein S6 kinase 1 and eukaryotic translation initiation factor 4E-binding protein 1, leading to accelerated cell growth and proliferation [8]. mTORC1 signaling is frequently over-activated in human cancers [11], but its precise mechanisms still require further clarification.

It has been well-recognized that mTORC1 is a positive regulator of angiogenesis [15, 16]. Hyperactivated mTORC1 upregulates hypoxia-inducible factor -1α at numerous levels, which in turn promotes angiogenesis by enhancing the transcription of pro-angiogenic factors, such as vascular endothelial growth factor (VEGF) and transforming growth factor -α [17]. It has also been demonstrated that the mTORC1 specific inhibitor rapamycin exerts anti-angiogenic properties by reducing the proliferation, migration, and tubular structure formation of endothelial cells [18]. We have previously reported that mTORC1 signaling activation promotes angiogenesis through the upregulation of brain-expressed X-linked 2 [19]. However, whether noncoding RNAs are involved in mTORC1-mediated angiogenesis remains unclear.

In this study, we showed that micro-RNA-130b-3p (miR-130b-3p) is involved in hyperactivated mTORC1-mediated angiogenesis and tumor growth. The transcription factor signal transducer and activator of transcription 3 (STAT3), serves as a downstream effector of mTORC1 and transcriptionally upregulates miR-130b-3p expression. Furthermore, miR-130b-3p exerts an angiogenesis-promoting role in mTORC1-activated cells by targeting muscleblind-like protein 1 (MBNL1)*.* In addition, MBNL1 depletion led to an increase in STAT3 activity. We suggest that the STAT3/miR-130b-3p/MBNL1 feedback loop is critical for mTORC1-mediated angiogenesis and tumor growth, and that it can be targeted for the treatment of cancers associated with dysregulated mTORC1 activity.

## Materials and methods

### Clinical specimens

A total of 60 paired head and neck squamous cell carcinoma (HNSCC) and adjacent normal mucosal (ANM) tissues were acquired at the First Affiliated Hospital of Anhui Medical University (Anhui, China) from 2014 to 2020. All recruited patients had not received chemotherapy, radiotherapy, or other antitumor therapies before surgery. The study was approved by the Research Ethics Committee of the First Affiliated Hospital of Anhui Medical University. All of the patients provided written informed consent before surgery. The patients’ detailed information is presented in Supplementary Table S1.

### Cell culture and treatment

All the mouse embryonic fibroblast (MEF) cell lines (including Tsc1 + / + , Tsc1 − / − , Tsc2 + / + , Tsc2 − / − , STAT3C-overexpressing Tsc2 + / + and the empty vector pBabe-transduced Tsc2 + / + MEFs) and NTC/T2 null (a cell line with potent tumorigenicity derived from a subcutaneous tumor formed by the injection of Tsc2 − / − MEFs in immunodeficient mice) cells have been described previously [20–22]. The HNSCC cell line FaDu, human umbilical vein endothelial cells (HUVECs), and HEK 293 T cells were obtained from the ATCC (VA, USA). The HNSCC cell line TU686 was obtained from the BeNa Culture Collection (Beijing, China). The HNSCC cell line TU212 and human normal oral keratinocyte (NOK) cells were obtained from Otwo Biotech (Shenzhen, China). HNSCC cell lines and NOK cells were cultured in 1640 medium (BOSTER, Wuhan, China) containing 10% fetal bovine serum (Gibco, CA, USA) (containing 100 U/mL of penicillin and 100 μg/mL of streptomycin); other cells were cultured in Dulbecco's Modified Eagle's Medium (DMEM) (BOSTER) with the same composition at 37 °C in a humidified incubator containing 5% CO_2_. For drug treatment, cells were seeded into 12-well plates at 30% − 40% density 24 h before treatment. The Dimethyl sulfoxide (DMSO) stocks of the agents used, including rapamycin, MHY-1485, and S3I-201, were diluted to appropriate concentrations with the cell culture medium. All the drugs were purchased from Selleck Chemicals (TX, USA).

### Lentivirus production and transduction

All of the lentiviral vectors were obtained from GeneChem (Shanghai, China), including the GV112 lentiviral shRNA expression vector targeting Raptor, Rictor, STAT3, MBNL1, and the control scrambled shRNẠ (shSc); GV209 and GV234 lentiviral expression plasmids that were used to increase and decrease miR-130b-3p, respectively; and GV341 lentiviral plasmid expressing mouse MBNL1 and the empty plasmid. The detailed information on the recombinant plasmids is presented in Supplementary Table S2.

Lentiviruses were generated by transfecting with either the recombinant vectors or control vectors together with packaging plasmids (psPAX2 and pVSVG) into HEK 293 T cells. Culture supernatants were collected and filtered after 48 h of transfection and then used to infect target cells at a multiplicity of infection of 10 to 20. Two days after infection, the cells were selected with 2 μg/mL of puromycin for 1 week following the manufacturer’s instructions.

### Immunoblotting

Immunoblotting analysis was performed as described previously [23]. In brief, cell or tissue lysates were resolved by NuPAGE 4–12% Bis–Tris gels (Life Technologies, CA, USA), transferred to PVDF membrane (Millipore, MA, USA), and then incubated with the primary and secondary antibodies. The specific protein bands in the membrane were visualized using Pierce™ ECL Western Blotting Substrate (Thermo Scientific, MA, USA) and ChemiScope 6100 (Clinx, Shanghai, China). All information regarding antibodies used in this study is provided in Supplementary Table S3.

### Quantitative real-time PCR

Total RNA from cells and tissues were isolated using TRIzol reagent (Life Technologies) according to the protocol provided by the manufacturer. For analysis of mRNA expression levels, first-strand cDNA synthesis was performed using the RevertAid™ First Stand cDNA Synthesis Kit (Thermo Scientific). Quantitative real-time PCR (qRT-PCR) detection of mRNA expression of MMP13, VEGF-C, BCL2, IL18, and MBNL1 was performed using SYBR Premix Ex Taq™ II (TaKaRa, Dalian, China). The expression of miR-130b-3p was detected using the Hairpin-itTM microRNAs qPCR Quantitation Kit (GenePharma, Shanghai, China) according to the producer’s instructions. qRT-PCR was performed on the LightCycler96 (Roche, Basel, Switzerland). β-actin or U6 served as an internal control. The primers used are listed in Supplementary Table S4.

### miRNA transfection

Cells were seeded into 12-well plates and allowed to reach 70% confluence before transfection. Transient transfection of mouse miR-130b-3p mimics, inhibitors, and the negative control (GenePharma) was conducted with Lipofectamine RNAiMax (Thermo Fisher Scientific) according to the protocol provided by the manufacturer. The sequences are listed as follows: miR-130b-3p mimics, 5'-CAGUGCAAUGAUGAAAGGGCAU-3'; mimics negative control (NC), 5'-UUCUCCGAACGUGUCACGUTT-3'; miR-130b-3p inhibitor, 5'-AUGCCCUUUCAUCAUUGCACUG-3'; and inhibitor negative control, 5'-CAGUACUUUUGUGUAGUACAA-3'.

### Reporter constructs and luciferase reporter assay

A 299-bp fragment of the mouse miR-130b promoter region (− 678/ − 976) embodying the wild-type STAT3 binding site was obtained by polymerase chain reaction (PCR) using mouse genomic DNA extracted from Tsc2 − / − MEFs and cloned into a pTAL-Luc vector (Clontech, CA, USA) for constructing miR-130b promoter luciferase reporter. A fragment of 330-bp MBNL1 3´-untranslated region (3´-UTR) containing the putative binding site for miR-130b-3p was generated by PCR and cloned into the p-miRGLO firefly luciferase vector (GenePharma). The potential STAT3 binding site on the promoter of the mouse miR-130b gene and the predicted miR-130b-3p target binding site in 3´-UTR of MBNL1 were mutated using the Q5 site-directed mutagenesis kit (NEB, MA, USA). The primers used to construct the recombinant plasmids are listed in Supplementary Table S5.

To investigate the effect of STAT3 activation on the miR-130b-3p promoter activity, HEK 293 T cells were cultured in triplicate to 80% confluence in 24-well plates and the promoter constructs (200 ng) were co-transfected with pBabe-STAT3C or the empty vector pBabe (200 ng) and the internal control plasmid pRL-TK (10 ng). Luciferase activity was detected with the Dual-Luciferase Reporter assay system (Promega, WI, USA) according to the manufacturer’s procedures.

To examine the effect of miR-130b-3p on the MBNL1 3´-UTR, HEK 293 T cells were transfected with 50 nM of miR-130b-3p mimics or NC-mimics, together with 100 ng of luciferase reporter plasmids (MBNL1-WT or MBNL1-Mut) using Lipofectamine 3000 (Invitrogen, CA, USA). Forty-eight hours after transfection, luciferase activities were measured as mentioned above.

### Immunofluorescence assay

MBNL1-overexpressing and control cells were seeded on fibronectin-coated glass coverslips in 24-well culture plates. After 24 h, the cells were fixed with 4% formaldehyde, treated with 1% Triton X-100 (Sigma-Aldrich, MO, USA) for permeabilization, and then blocked with 2% bovine serum albumin. The coverslips were incubated in a primary antibody overnight and followed with a CY3-conjugated secondary antibody (Cell Signaling Technology, MA, USA) for 1 h. An LSM880 + Airyscan confocal laser scanning microscope (Carl Zeiss, Oberkochen, Germany) was used to capture images.

### Chromatin immunoprecipitation

Chromatin immunoprecipitation (ChIP) assay was performed using a SimpleChIP® Plus Enzymatic Chromatin IP kit (Cell Signaling Technology) as described previously [22]. In brief, cells were cross-linked by 1% formaldehyde for 10 min, cracked with the sodium dodecyl sulfate lysis buffer, followed by ultrasonication for 50 min, and then incubated with an anti-phosphor-STAT3 Tyr^705^ (p-STAT3) antibody or an anti-H3K4me3 antibody (#ab213224, Abcam, Cambridge, UK) overnight. The immunoprecipitated DNA was purified and analyzed using PCR or qRT-PCR with specific primers. The primer sequences are listed in Supplementary Table S6.

### Tube formation assay

Cell-derived conditioned medium (CM) was prepared as described previously [23]. Briefly, stably transfected cells were seeded in a 10-cm dish and incubated for 48 h, after which the medium was removed and replaced by a fresh serum-deprived medium. After 24 h of incubation, the CM was collected, filtered, and then concentrated by ultrafiltration using Amicon Ultra 10 K centrifugal filters (Millipore). The CM was stored at − 80 °C until use.

For the tube formation assay, a total of 150 µL of Matrigel (Corning, NY, USA) was added to a 48-well plate and incubated at 37 °C for 30 min. Then, HUVECs (3.5 × 10^4^) in 300 µL of prepared CM were added to each well and incubated at 37 °C in 5% CO_2_. After incubation for 12 h, bright-field images were recorded using a microscope and analyzed using WimTube (https://www.wimasis.com/en/WimTube, Wimasis GmbH, Munich, Germany).

### Chicken chorioallantoic membrane assay

Pathogen-free fertilized chicken eggs were purchased from Jinan SAIS Poultry Company (Shandong, China) and handled as described previously [23]. Briefly, on embryonic developmental days 8, sterile gelatin sponges mixed with 20 μL of cell suspension containing 5 × 10^6^ cells were deposited on chicken chorioallantoic Membrane (CAM). On embryonic developmental days 14–17, the CAM was separated, fixated, and photographed. The number of blood vessels that converged toward the implant was counted by two blind observers.

### Enzyme-linked immunosorbent assay

The levels of secreted VEGF-C in cell-free supernatant of MBNL1-overexpressing cells and the control cells were quantified using a mouse VEGF-C ELISA Kit (Novus Biologicals, CO, USA) according to the manufacturer’s instructions.

### RNA immunoprecipitation assay

The RNA immunoprecipitation assay experiments were performed with a BersinBio™ RIP Kit (BersinBio, Guangdong, China) according to the manufacturer’s instructions. Briefly, cells were collected and lysed with RIP lysis buffer and then incubated with magnetic protein A beads coupled with anti-Argonaute protein 2 (AGO2) antibody (#186,733, Abcam) for 6 h at 4 °C. Thereafter, the RNA was purified, and the levels of miR-130b-3p and MBNL1 were analyzed using qRT-PCR. IgG served as a negative control. The primers used are listed in Supplementary Table S4.

### Animal experiments

BALB/c-Nude mice and NOD/SCID mice were purchased from GemPharmatech (Nanjing, China). All animals were maintained in strict accordance with the guidelines of the Animal Center of Anhui Medical University, and all animal experimental procedures were approved by the Experimental Animal Ethical Committee of Anhui Medical University.

For in vivo xenograft assay, Nude mice were randomly assigned into groups (five mice per group) to receive respective treatments. In the right anterior armpit of the mice, 4 × 10^6^ genetically engineered cells in 0.2 mL of DMEM were inoculated subcutaneously. Tumor growth was detected every 3 days, and tumor volume was calculated using the following equation: Volume = (length × width^2^)/2(mm^3^).

To establish the patient-derived xenograft (PDX) models, tumor tissues from an HNSCC patient were processed after tumor resection. The tumor samples were cleaned, then cut into small pieces with diameters of approximately 3–5 mm, and subcutaneously implanted into the flanks of NOD/SCID mice. The successfully established PDX model was referred to as passage 1 (P1). When the tumor volume reached approximately 1,000 mm^3^, mice were sacrificed, and the tumors were passaged and expanded for two more generations (named P2 and P3) using the same previously described procedure as with Nude mice. The P3 xenografts were treated intratumorally with either antagomiR-130b-3p (a 2´-OMe + 5´-chol–modified miR-130b-3p inhibitor) or the scramble control antagomiR-NC (GenePharma) at a concentration of 10 nmol/50 μL once every three days when tumors reached a volume of 70–100 mm^3^. On day 28 after treatment, animals were sacrificed, and tumors were collected for further analysis.

### Immunohistochemistry analysis

Paraffin-embedded tumor tissues were cut into 4-μm slices. The histological sections were stained with antibodies against p-S6, CD31, p-STAT3, and MBNL1, according to the manufacturer’s protocols. For the determination of p-S6 immunoreactivity in HNSCC samples, immunohistochemistry analysis (IHC) staining was scored on the basis of the intensity of staining and the proportion of positive cells. The blinded review was performed by two pathologists.

### Bioinformatics analysis

RNA sequencing data from HNSCC (*n* = 569), breast invasive carcinoma (BRCA; *n* = 1,207), esophageal carcinoma (ESCA; *n* = 200), liver hepatocellular carcinoma (LIHC; *n* = 425), lung adenocarcinoma (LUAD; *n* = 567), and skin cutaneous melanoma (SKCM; *n* = 452) patients were obtained from The Cancer Genome Atlas (TCGA) (http://cancergenome.nih.gov/). Gene set enrichment analysis (GSEA) was performed to examine the enrichment of mTORC1 positively regulated gene sets in miR-130b-3p-high and miR-130b-3p-low HNSCC, BRCA, ESCA, LIHC, LUAD, and SKCM cancer tissues, according to methods described previously [22].

### Statistical analysis

Data were analyzed using Student’s t-test (two-tailed) or one-way analysis of variance (ANOVA) as appropriate, with GraphPad Prism 6.0 software. The association of miR-130b-3p and p-S6 was analyzed using Pearson’s correlation analysis. *P* < 0.05 is considered statistically significant.

## Results

### Hyperactivated mTORC1 upregulates miR-130b-3p

**Fig. 1:**
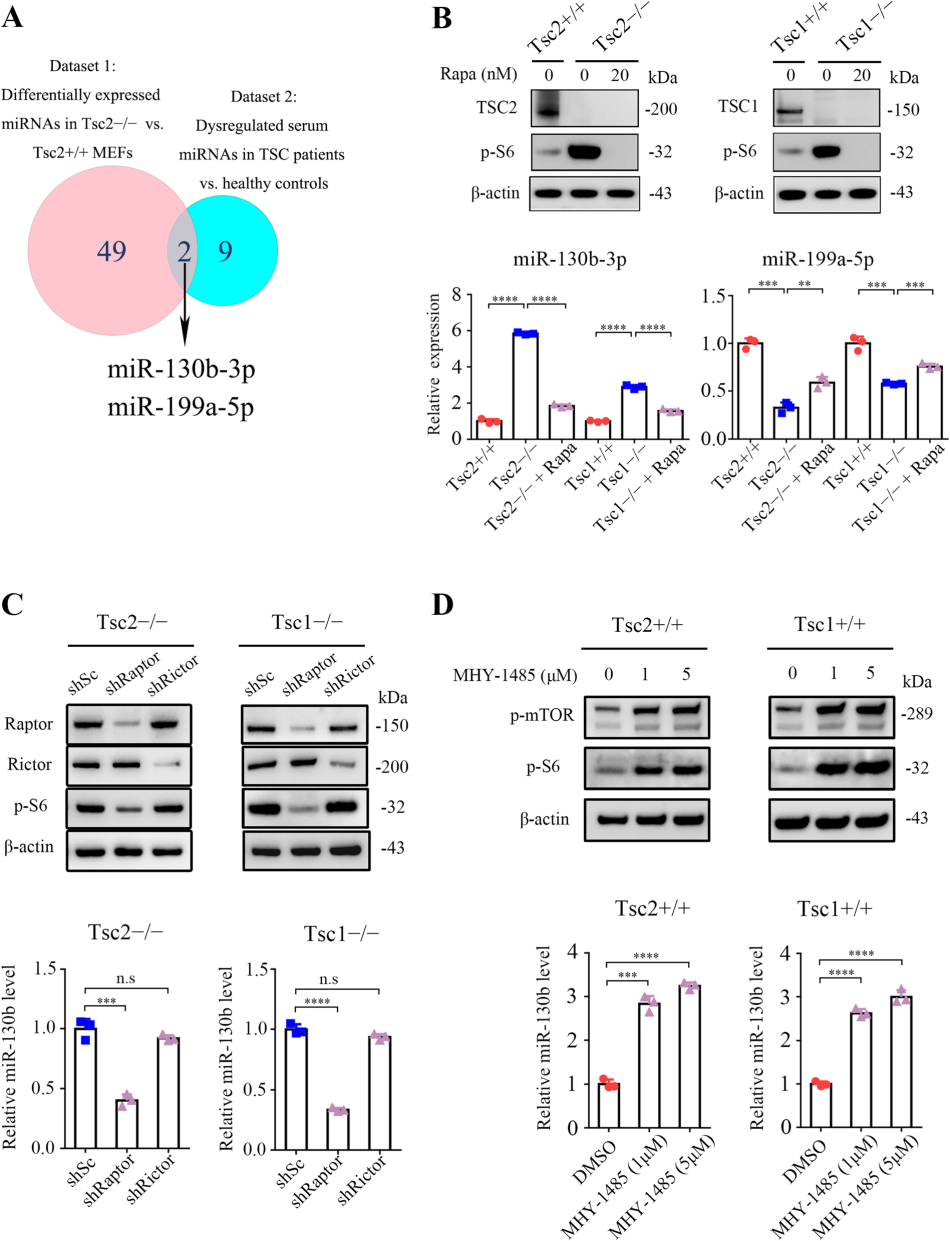
mTORC1 upregulates the expression of miR-130b-3p. **A** Venn diagram analysis of differentially expressed miRNAs in two datasets. Dataset 1 represents differentially expressed miRNAs between Tsc2 − / − and Tsc2 + / + MEFs. Dataset 2 represents dysregulated serum miRNAs between TSC patients and healthy controls. **B** Tsc2 + / + , Tsc2 − / − , rapamycin (20 nM, 24 h) treated Tsc2 − / − , Tsc1 + / + , Tsc1 − / − and rapamycin (20 nM, 24 h) treated Tsc1 − / − MEFs. **C** Tsc2 − / − or Tsc1 − / − MEFs were infected with lentiviruses harboring vectors encoding Raptor shRNA (shRaptor), Rictor (shRictor) or the control scrambled shRNA (shSc). **D** Tsc2 + / + or Tsc1 + / + MEFs were treated with or without MHY-1485 (1 or 5 μM) for 24 h. **B-D** Cell lysates were subjected to immunoblotting with the indicated antibodies (up panels); miR-130b-3p level was analyzed by qRT-PCR (low panels). Data indicate mean ± SD of triplicate samples. ** *P* < 0.01; *** *P* < 0.001; **** *P* < 0.0001; n.s indicates no significant difference

Because the TSC1/TSC2 complex is the principal suppressor of mTORC1 signaling and hyperactivated mTORC1 is the main reason for tumor formation in TSC disease [24], Tsc1 − / − or Tsc2 − / − MEFs and TSC samples are excellent models for the study of mTORC1 signaling. In our previous study, 51 differentially expressed miRNAs (13 upregulated and 38 downregulated) in Tsc2 − / − MEFs as compared with Tsc2 + / + MEFs (fold change > 2) were identified [25]. To examine effectively functional miRNAs downstream of mTORC1, a Venn analysis was performed using the aforementioned 51 miRNAs and miRNAs that are abnormally expressed in the serum of patients with TSC [26]. Two miRNAs were screened, including miR-130b-3p and miR-199a-5p (Fig. 1A). The qRT-PCR analysis confirmed that miR-130b-3p levels were significantly increased and the miR-199a-5p level was downregulated in Tsc1- or Tsc2-null MEFs as compared with their corresponding control cells, and their levels were reversed by mTORC1 inhibition with rapamycin treatment (Fig. 1B). MiR-130b-3p was chosen for further study because the degree of its expression change is larger than that of miR-199a-5p.

To further confirm that it is indeed mTORC1 that mediates the positive regulation of miR-130b-3p downstream of the TSC1/TSC2 complex, miR-130b-3p levels in Raptor (a specific component of mTORC1) or Rictor (a specific component of mTORC2)-knockdown Tsc2 − / − MEFs were examined. As indicated in the left panels of Fig. 1C, cells infected with Raptor shRNAs exhibited decreased miR-130b-3p levels, whereas Rictor shRNAs had little effect on miR-130b-3p expression. A consistent result was obtained in Tsc1 − / − MEFs after either Raptor or Rictor depletion (Fig. 1C, right panels). In addition, mTORC1 activation by treatment of MHY-1485 led to the upregulation of miR-130b-3p in Tsc2 + / + or Tsc1 + / + MEFs (Fig. 1D). Together, these data suggest that hyperactivated mTORC1 upregulates miR-130b-3p expression.

### MiR-130b-3p promotes angiogenesis and tumor growth

**Fig. 2:**
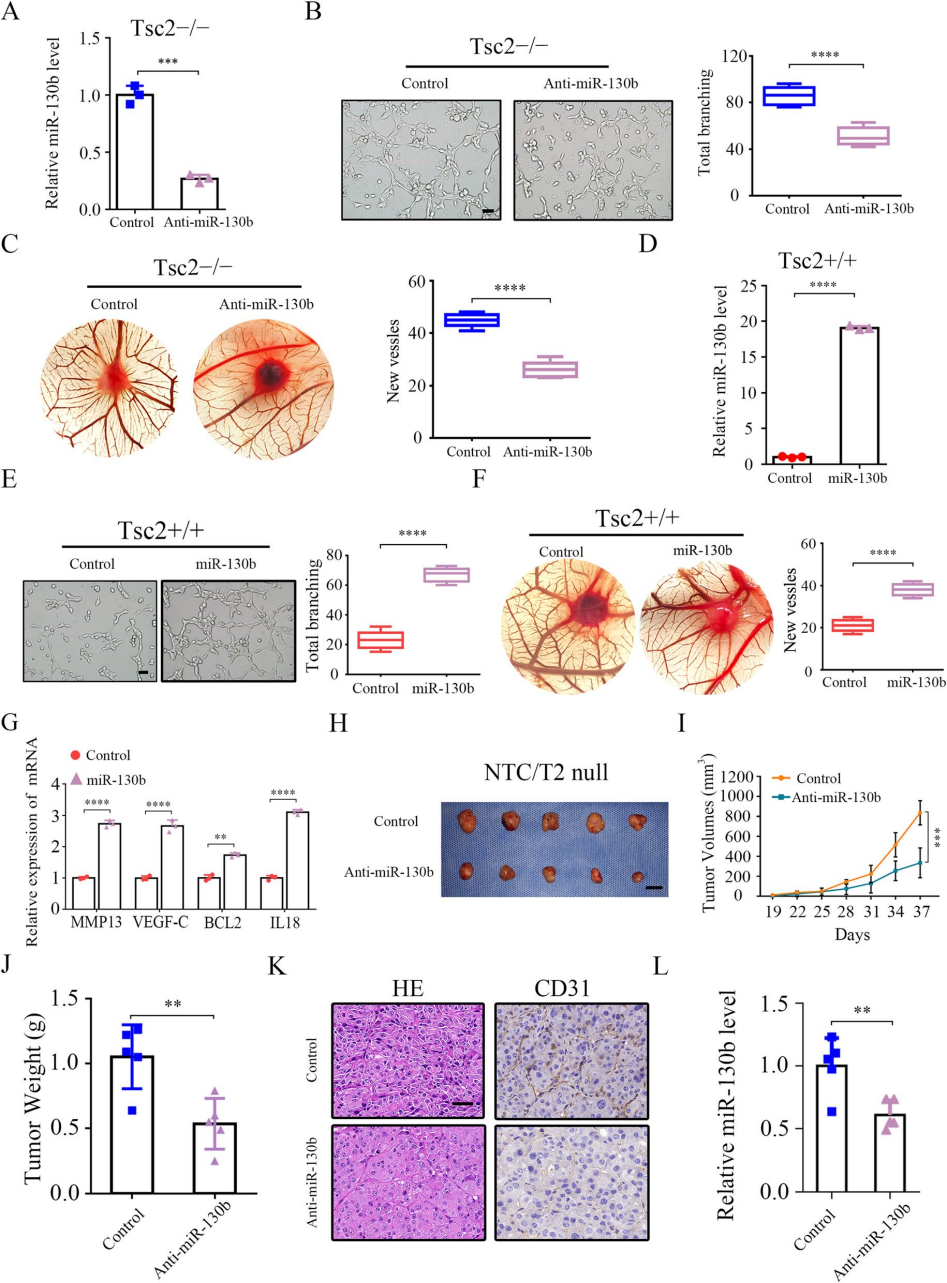
miR-130b-3p positively modulates the pro-angiogenic ability of mTORC1-activated cells. **A-C** Tsc2 − / − MEFs were transduced with lentivirus expressing an antisense sequence against miR-130b-3p (anti-miR-130b) or a scrambled sequence (control). **D-G** Tsc2 + / + MEFs were infected either with a lentivirus encoding miR-130b-3p (miR-130b) or a scrambled miRNA sequence (control). **A** and **D** The expression of miR-130b-3p was determined by qRT-PCR. **B**, **C**, **E**, and **F** The effect on angiogenesis was tested by tube formation assay (**B** and **E**, scale bar, 50 μm) and CAM assay (**C** and **F**). Representative images (left panels) and quantifications (right panels). **G** mRNA levels of the indicated angiogenesis-related genes were measured by qRT-PCR. **H–L** Tumor growth of mice subcutaneously injected with NTC/T2 null cells stably transduced with anti-miR-130b-3p or control lentiviruses. *N* = 5 for each group. (H) Tumor pictures. Scale bar, 1 cm. **I** Tumor growth curves. **J** Tumor weight. **K** Representative HE and IHC staining for CD31 of the indicated tumor tissues. Scale bar, 50 μm. **L** miR-130-3p levels in the indicated tumor tissues were determined by qRT-PCR. Data indicate mean ± SD of 3–5 replicates. ** *P* < 0.01; *** *P* < 0.001; **** *P* < 0.0001

Because miR-130b-3p has been linked to tumor angiogenesis in various types of cancer, and mTORC1 is a critical activator of angiogenesis [15, 16, 27, 28], we were greatly interested in investigating whether miR-130b-3p plays a vital role in mTORC1-mediated angiogenesis and tumor progression. First, Tsc2 − / − and Tsc1 − / − MEFs were stably transfected with antisense of miR-130b-3p (anti-miR-130b) (Fig. 2A and Supplementary Fig. S1A). Subsequently, in vitro capillary tube formation assay was performed. It was observed that HUVECs cultured with the CM derived from the miR-130b-3p suppressed cell lines developed less capillary-like structures and branch points, implying the pro-angiogenesis function of miR-130b-3p (Fig. 2B and Supplementary Fig. S1B). Moreover, the CAM assay also revealed that miR-130b-3p suppression strongly attenuated the formation of new microvessels (Fig. 2C). Conversely, miR-130b-3p overexpression enhanced the angiogenic capacity of Tsc2 + / + MEFs (Fig. 2D − F). Consistently, the angiogenesis-related genes, including MMP13, VEGF-C, BCL2, and IL18, were found to be upregulated in miR-130b-3p-overexpressing Tsc2 + / + MEFs (Fig. 2G). In summary, these findings suggest that miR-130b-3p could enhance the pro-angiogenic capacity of mTORC1-activated cells.

To further investigate the in vivo role of miR-130b-3p, NTC/T2 null cells transfected with a lentiviral vector encoding anti-miR-130b-3p or a scrambled sequence were subcutaneously injected into the right anterior armpit of Nude mice (Supplementary Fig. S1C − D), and then tumor growth was monitored. As depicted in Fig. 2H − J, the anti-miR-130b-3p group, compared with the control group, showed a significant reduction in tumor volume and weight. Furthermore, IHC analysis demonstrated that tumor tissues derived from anti-miR-130b-3p-expressing cells exhibited much weaker staining for the angiogenic marker CD31 than those in the control counterpart (Fig. 2K). Decreased miR-130b-3p expression in the tumor samples derived from animals bearing anti-miR-130b-3p-expressing cell xenografts was confirmed by qRT-PCR (Fig. 2L). Therefore, miR-130b-3p positively regulates angiogenesis and tumor growth driven by mTORC1 activation.

### mTORC1 upregulates miR-130b-3p through STAT3 activation

**Fig. 3:**
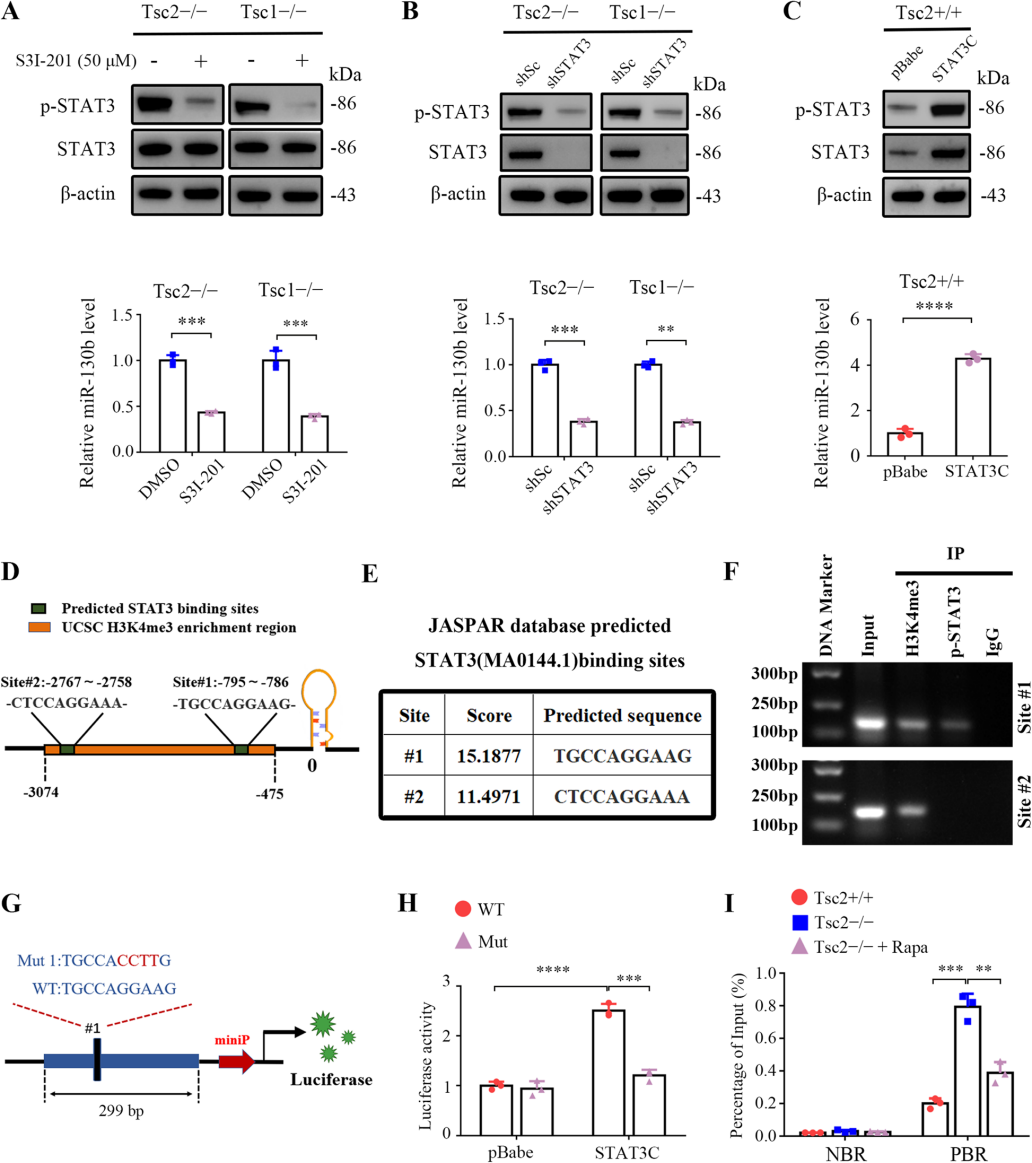
mTORC1 upregulates miR-130b-3p through activation of STAT3. **A** Tsc2 − / − or Tsc1 − / − MEFs were treated with 50 μM S3I-201 or DMSO for 24 h. **B** Tsc2 − / − MEFs or Tsc1 − / − MEFs were infected with lentiviruses encoding STAT3 shRNA (shSTAT3) or the control shRNA (shSc). **C** Tsc2 + / + MEFs were transduced with the retroviruses for STAT3C in pBabe or its control vector pBabe. **A-C** Cell lysates were subjected to western blot analysis using the indicated antibodies (up panels). The level of miR-130b-3p was detected by qRT-PCR (low panels). **D** and **E** Schematic illustration of the promoter region of miR-130b (**D**) and the two conserved STAT3-binding sites predicted by the JASPAR database (**E**). **F** The enrichment of H3K4me3 and STAT3 in the promoter of miR-130b was analyzed by ChIP-PCR assay. **G** Schematic illustration of the construction of miR-130b promoter luciferase reporters containing a region around site #1 with the STAT3 binding site intact (WT) or mutated (Mut). **H** HEK 293 T cells were co-transfected with WT-Luc or Mut-Luc plus pBabe-STAT3C or control pBabe and the internal control plasmid pRL-TK. Relative luciferase activity was detected 24 h after transfection. **I** Tsc2 + / + , Tsc2 − / − , and rapamycin (20 nM, 24 h) treated Tsc2 − / − MEFs were subjected to ChIP analysis with antibodies to p-STAT3 or control rabbit IgG. qRT-PCR was performed to amplify regions surrounding the putative STAT3 binding region (PBR) and a nonspecific STAT3 binding region (NBR). The data were plotted as the ratio of immunoprecipitated DNA to total input DNA. Error bars indicate mean ± SD of triplicate samples. **P < 0.01; *** *P* < 0.001; **** *P* < 0.0001

Because the transcription factor STAT3 is one of the most vital downstream effectors of mTORC1 and is a vital driver of angiogenesis [20, 29], we investigated whether STAT3 participates in the regulation of miR-130b-3p expression downstream of mTORC1. We first evaluated the effect of S3I-201, a specific STAT3 inhibitor, on miR-130b-3p expression. As indicated in Fig. 3A, S3I-201 treatment led to a marked reduction of miR-130b-3p in Tsc2 − / − and Tsc1 − / − MEFs. Similarly, knockdown of STAT3 with lentivirus mediated STAT3 shRNA led to significantly decreased miR-130b-3p expression in both Tsc2 − / − and Tsc1 − / − MEFs (Fig. 3B). In contrast, overexpression of a constitutively activated STAT3 (STAT3C) resulted in dramatically upregulated miR-130b-3p expression in Tsc2 + / + MEFs (Fig. 3C). Therefore, mTORC1 upregulates miR-130b-3p through STAT3 activation.

Next, we further analyzed whether STAT3 binds to the miR-130b promoter region to activate its expression. H3K4me3 is a highly conserved histone modification and a hallmark of active genes distributed along with promoter regions [30, 31]. By retrieving the UCSC Genome Browser (http://genoime.ucsc.edu/), a genomic region located from approximately − 3074 to − 475 bp ahead of miR-130b stem-loop was determined to have enrichment of the H3K4me3 histone modification (Fig. 3D). Further analysis via the JASPAR database (http://jaspar.genereg.net/) predicted two conserved STAT3-binding sites in this H3K4me3-enriched region (Fig. 3E). ChIP assay indicated that STAT3 was only enriched in site #1 (Fig. 3F), suggesting that this site may have critical importance for the transcription of miR-130b. Furthermore, miR-130b promoter luciferase reporters containing a region around site #1 were constructed (Fig. 3G). STAT3C overexpression significantly increased the luciferase activity of the reporter, whereas the enhanced transcriptional activity was attenuated when the putative STAT3 binding site was mutated (Fig. 3G and H). Moreover, the ChIP assay displayed that the recruitment of the STAT3 protein to site #1 was significantly enhanced in Tsc2 − / − MEFs in comparison with Tsc2 + / + MEFs, and the enrichment was attenuated with the addition of rapamycin (Fig. 3I). On the basis of these results, we conclude that STAT3 transcriptionally elevates miR-130b-3p through direct binding with its promoter.

### MBNL1 is a direct target gene of miR-130b-3p

**Fig. 4:**
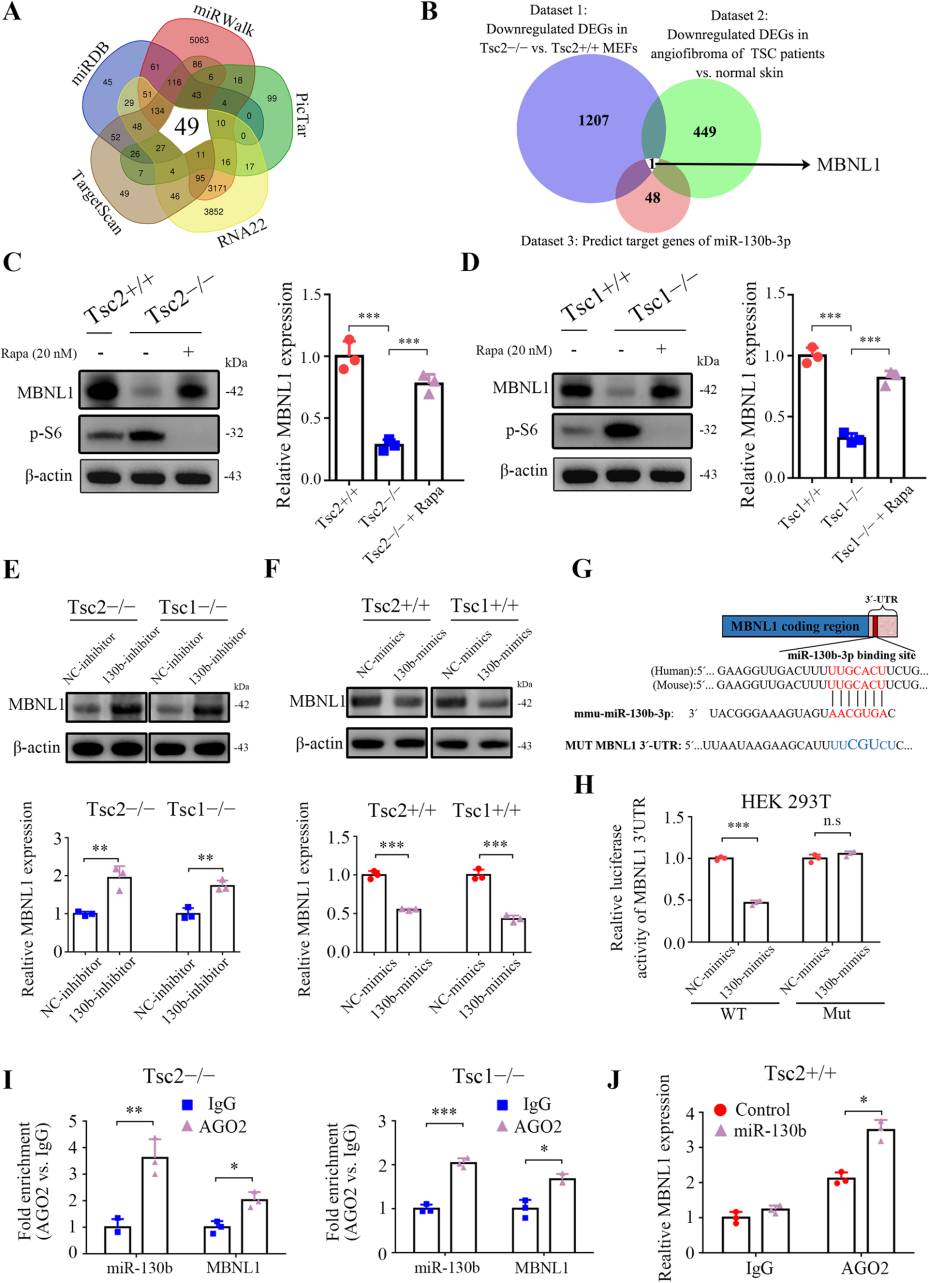
MBNL1 is a direct target of miR-130b-3p. **A** Screening for miR-130b-3p target genes by prediction softwares TargetScan, RNA22, PicTar, miRDB and miRWalk. **B** Venn diagram of DEGs in three datasets. Dataset 1 represents downregulated DEGs in Tsc2 − / − vs. Tsc2 + / + MEFs. Dataset 2 represents decreased DEGs in AF of TSC patients vs. normal skin. Dataset 3 represents predicted target genes of miR-130b-3p. **C** Tsc2 + / + , Tsc2 − / − and rapamycin (20 nM, 24 h) treated Tsc2 − / − MEFs. **D** Tsc1 + / + , Tsc1 − / − and rapamycin (20 nM, 24 h) treated Tsc1 − / − MEFs. **C** and **D** Cell lysates and total RNA were subjected to western blot (left panels) and qRT-PCR (right panels) analyses. **E** Tsc2 − / − and Tsc1 − / − MEFs were transfected with the miR-130b-3p inhibitor or negative control (NC-inhibitor). **F** Tsc2 + / + and Tsc1 + / + MEFs were transfected with the miR-130b-3p mimics or negative control (NC-mimics). **E** and **F** MBNL1 and miR-130b-3p levels in the indicated cells were examined by western blot (up panels) or qRT-PCR (low panels). **G** Sequence alignment of the predicted miR-130b-3p binding site within the mouse MBNL1 3´-UTR and its mutated sequence for luciferase reporter assay. **H** Luciferase reporter assay was performed in HEK 293 T cells that were co-transfected with miR-130b-3p mimics or NC-mimics together with reporter vectors containing MBNL1 3´-UTR or mutated MBNL1 3´-UTR. Relative luciferase activities are presented. **I** RIP assay was performed using antibodies against AGO2 or IgG and the enrichment of miR-130b-3p and MBNL1 were detected by qRT-PCR in Tsc2 − / − or Tsc1 − / − MEFs. **J** RIP assay was performed using AGO2 or IgG antibodies to estimate the enrichment of MBNL1 in Tsc2 + / + MEFs infected with lentivirus expressing miR-130b-3p or a control lentivirus. Data indicate mean ± SD of triplicate samples. * *P* < 0.05; ** *P* < 0.01; *** *P* < 0.001; n.s indicates no significant difference

The putative target genes of miR-130b-3p were predicted by the following bioinformatics tools: TargetScan, picTar, RNA22, miRDB, and miRWalk. The intersections were retrieved by starBase V2.0, and 49 genes were found to be potential targets (Fig. 4A and Supplementary Table S7). To further screen target genes, the Venn analysis of these predicted target genes with downregulated differentially expressed genes in Tsc2 − / − MEFs as compared with Tsc2 + / + MEFs and decreased differentially expressed genes in angiofibroma (AF) of TSC patients vs. normal skin obtained only one candidate, MBNL1 [32, 33] (Fig. 4B, Supplementary Tables S8 and S9). qRT-PCR and western blot analyses confirmed that loss of TSC1 or TSC2 led a significantly downregulated MBNL1 expression, whereas MBNL1 expression was upregulated in response to mTORC1 inhibition (Fig. 4C and D). Furthermore, transfection of miR-130b-3p inhibitor led to upregulated MBNL1 expression in Tsc2 − / − or Tsc1 − / − MEFs (Fig. 4E). In contrast, transfection of miR-130b-3p mimics had the opposite effect in Tsc2 + / + or Tsc1 + / + MEFs (Fig. 4F). Therefore, mTORC1 downregulates MBNL1 through the upregulation of miR-130b-3p.

To further assess whether MBNL1 is a direct target of miR-130b-3p, a luciferase activity assay was performed. A fragment of the wild-type 3´-UTR (MBNL1-WT) and a fragment of the mutant 3´-UTR (MBNL1-Mut) were cloned into the p-miRGLO firefly luciferase vector (Fig. 4G). HEK 293 T cells were co-transfected with MBNL1-WT or MBNL1-Mut, miR-130b-3p mimics, or NC-mimics. As indicated in Fig. 4H, the relative luciferase activity of the reporter containing the WT 3´-UTR was significantly decreased when co-transfected with miR-130b-3p mimics. Conversely, the luciferase activity of the mutant 3´-UTR was similar between the miR-130b-3p mimics and NC-mimics.

Because miRNAs exert their roles mainly through binding with AGO2 to form an RNA-induced silencing complex [34], anti-AGO2 RIP assays were performed to further confirm our findings. RIP demonstrated that AGO2, compared with IgG, was able to enrich both miR-130b-3p and MBNL1 as compared with IgG (Fig. 4I). Moreover, it was observed that there was a dramatic increase in the levels of MBNL1 3´-UTR in anti-AGO2 RIP compared with anti-IgG RIP in response to miR-130b-3p overexpression (Fig. 4J). Collectively, the aforementioned data suggested that MBNL1 is a direct downstream target of miR-130b-3p.

### miR-130b-3p promotes angiogenesis and tumor progression by targeting MBNL1

**Fig. 5:**
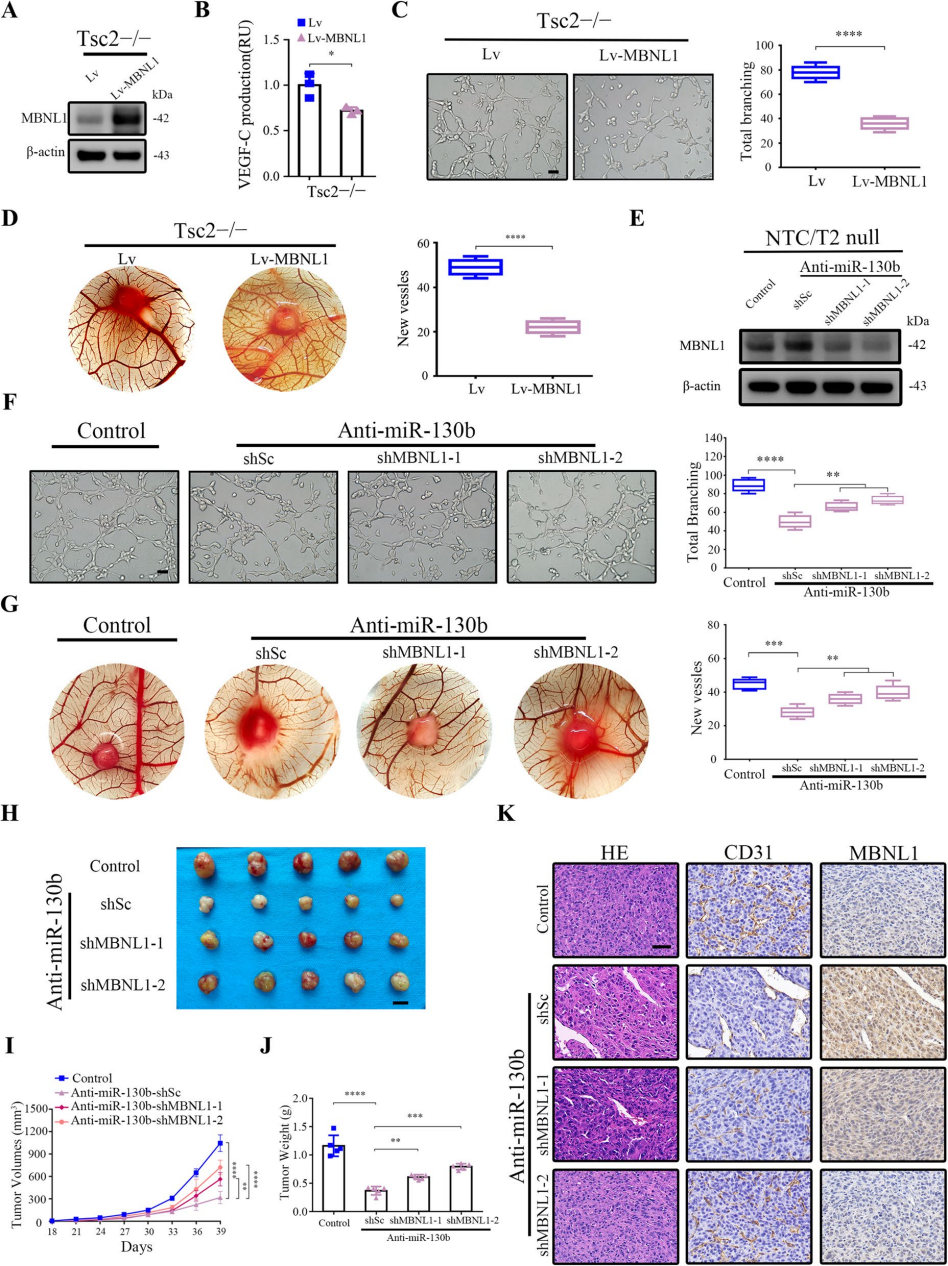
miR-130b-3p promotes angiogenesis via targeting MBNL1. **A-D** Tsc2 − / − MEFs were infected with lentiviruses harboring control vector (Lv) or MBNL1 overexpression constructs (Lv-MBNL1). **A** The expression of MBNL1 was determined by western blot. **B** Cell culture supernatants from the cells were analyzed for VEGF-C by ELISA. (RU, relative unit). **C** and **D** The effect on angiogenesis of was detected by tube formation assay (**C**, scale bar, 50 μm) and CAM assay (**D**). **E–G** anti-miR-130b-3p-expressing NTC/T2 null cells were infected with lentivirus harboring shRNAs targeting MBNL1 (shMBNL1-1 and shMBNL1-2) or a control shRNA (shSc). **E** Cell lysates were subjected to immunoblotting with the indicated antibodies. **F** and **G** The angiogenic abilities of the indicated cells were detected by tube formation assay (**F**, scale bar, 50 μm) and CAM assay (**G**). Representative images (left panels) and quantifications (right panels). **H–K** The indicated cells were inoculated subcutaneously into nude mice (*n* = 5), and tumor growth was monitored. **H** Tumor pictures. Scale bar, 1 cm. **I** Tumor volumes, **J** Tumor weight. **K** Tumor tissues were subjected to HE and IHC staining. Scale bar, 50 μm. Data indicate mean ± SD of 3–5 replicates. * *P* < 0.05; ** *P* < 0.01; *** *P* < 0.001; **** *P* < 0.0001

To examine the potential significance of MBNL1 in angiogenesis, we ectopically expressed MBNL1 with lentivirus in Tsc2 − / − and Tsc1 − / − MEFs (Fig. 5A and Supplementary Fig. S2A). Enzyme-linked immunosorbent assay (ELISA) revealed that the VEGF-C levels were significantly lower in the MBNL1-overexpressing groups (Fig. 5B and Supplementary Fig. S2B). In vitro tube formation assays demonstrated that the HUVECs treated with CM derived from the MBNL1 transfectants developed less capillary-like structures than did cells treated with CM derived from empty vector transfectants (Fig. 5C and Supplementary Fig. S2C). Moreover, the CAM assay also revealed that MBNL1 overexpression dramatically suppressed the formation of new microvessels (Fig. 5D and Supplementary Fig. S2D). The in vivo function of MBNL1 was further evaluated using a subcutaneous xenograft tumor model. As indicated in Supplementary Fig. S2E − I, MBNL1 overexpression attenuated tumor angiogenesis (as assessed by CD31 index) and xenograft growth. Together, these data revealed that MBNL1 exerts an anti-angiogenic activity.

To investigate whether the pro-angiogenic effect of miR-130b-3p is dependent on MBNL1, a lentiviral vector expressing shRNAs for MBNL1 was transduced to anti-miR-130b-3p-expressing Tsc2 − / − MEFs or NTC/T2 null cells. The knockdown efficiencies in the transformed cell lines were detected by western blot (Fig. 5E and Supplementary Fig. S3A). MBNL1 knockdown partially rescued the inhibitory effect of miR-130b-3p suppression on angiogenesis (Fig. 5F, G and Supplementary Fig. S3B). In contrast, ectopically expressed MBNL1 in miR-130b-3p-overexpressing Tsc2 + / + MEFs partially attenuated the pro-angiogenic effect of miR-130b-3p (Supplementary Fig. S3C and D). The mediation effect of MBNL1 on the miR-130b-3p-regulated angiogenesis and tumor growth was further confirmed using a subcutaneous xenograft tumor model. Consistent with the forementioned results, MBNL1 depletion rescued the suppressive effects of miR-130b-3p inhibition on xenograft growth and tumor angiogenesis (Fig. 5H − K). Taken together, these data revealed that miR-130b-3p promotes angiogenesis and tumor growth at least partially through suppression of MBNL1 expression.

### The mTORC1/STAT3/miR-130b-3p/MBNL1 signaling pathway exists in human cancers

**Fig. 6:**
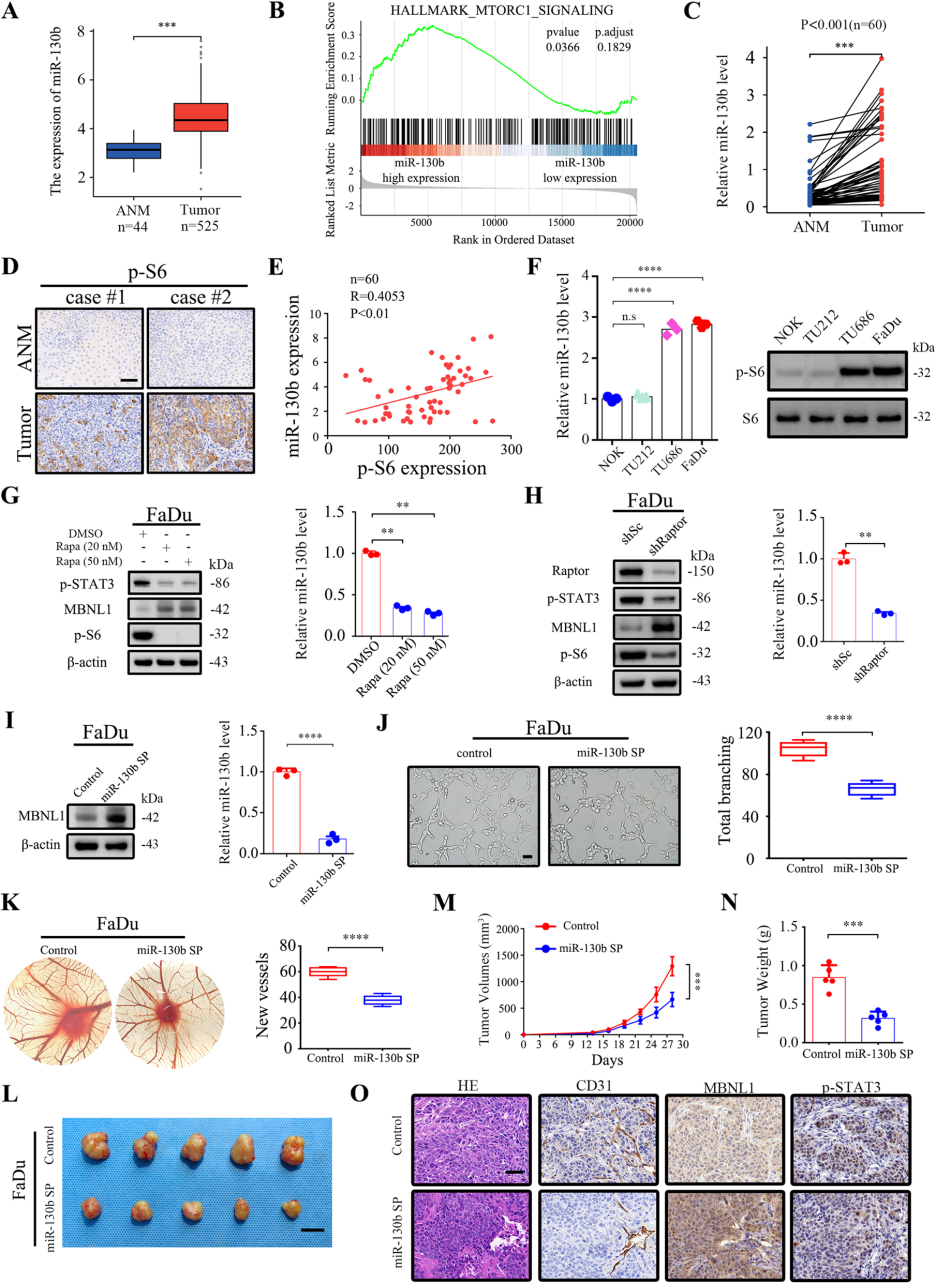
The mTORC1/STAT3/miR-130b-3p/MBNL1 signaling pathway exists in human cancer. **A** Expression analysis of miR-130b-3p in HNSCC and ANM tissues using TCGA. **B** GSEA analysis comparing the gene sets positively regulated by mTORC1 signaling in miR-130b-3p-high and miR-130b-3p-low HNSCC patients based on TCGA datasets. **C** miR-130b-3p levels were measured by qRT-PCR using the paired ANM tissues and HNSCC tissues (*n* = 60). **D** Representative IHC images of p-S6 staining from HNSCC tissues. Scale bar, 50 μm. **E** The correlation between p-S6 and miR-130b-3p expression of HNSCC tissues. **F** p-S6 and miR-130b-3p levels of the indicated cells were analyzed by qRT-PCR and western blot, respectively. **G** FaDu cells were treated with rapamycin (20 nM or 50 nM) for 24 h. **H** FaDu cells were infected with lentivirus expressing shRNAs targeting Raptor (shRaptor) or a control shRNA (shRNA). **I** FaDu cells were transduced with lentiviruses expressing sponge sequences of miR-130b-3p (miR-130b SP) or a control lentivirus. **G-I** Cell lysates were subjected to immunoblotting with the indicated antibodies (left panels), the expression of miR-130b-3p was detected by qRT-PCR (right panels). **J** and **K** The pro-angiogenic effect of miR-130b-3p was detected by tube formation (**J**, scale bar, 50 μm) and CAM assays (**K**). Representative images (left panels) and quantifications (right panels) are shown. (L-O) miR-130b SP-expressed FaDu cells and the control cells were inoculated subcutaneously into nude mice, and tumor growth was monitored. **L** Tumor pictures. Scale bar, 1 cm. **M** Tumor growth curves. **N** Tumor weight. **O** Representative HE and IHC staining of the indicated tumor tissues. Scale bar, 50 μm. Data indicate mean ± SD of 3–5 replicates. ** *P* < 0.01; *** *P* < 0.001; **** *P* < 0.0001; n.s indicates no significant difference

TCGA database was used to evaluate the clinical relevance of this newly discovered mTORC1 regulation of miR-130b-3p in human cancers. As indicated in Fig. 6A, the analysis of TCGA dataset showed that miR-130b-3p was remarkably upregulated in HNSCC tissues compared with ANM tissues. GSEA further demonstrated that the genes positively regulated by mTORC1 signaling were enriched in miR-130b-3p-high expression groups in HNSCC (Fig. 6B). Similarly, miR-130b-3p was also significantly upregulated and positively correlated with mTORC1 signaling in multiple human cancers, such as SKCM, LIHC, LUAD, BRCA, and ESCA (Supplementary Fig. S4A and B). Furthermore, through analysis of 60 paired human HNSCC lesions and the corresponding ANM tissues, it was confirmed that both miR-130b-3p and p-S6 were upregulated in tumor tissues, and the expression level of miR-130b-3p was positively correlated with mTORC1 activity (Fig. 6C − E). Therefore, miR-130b-3p expression positively correlated with mTORC1 activity in human cancer tissues, and it may play a critical role in mTORC1-mediated tumorigenesis.

Next, miR-130b-3p and p-S6 levels were detected in various HNSCC cell lines. As compared with NOK cells, HNSCC cells (FaDu and TU686) exhibited high levels of mTORC1 activity and miR-130b-3p (Fig. 6F). FaDu cells were chosen for further study. As indicated in Fig. 6G and H, mTORC1 inhibition by either pharmacological or genetic strategies led to the downregulation of p-STAT3 and miR-130b-3p. As expected, MBNL1 was substantially upregulated in response to mTORC1 suppression. Consistent with Tsc1- or Tsc2-null MEFs, miR-130b-3p inhibition through expressing sponge sequences targeting miR-130b-3p upregulated the MBNL1 expression and suppressed angiogenesis as evaluated by tube formation and CAM assays (Fig. 6I − K). Inhibition of the mTORC1/miR-130b-3p axis in TU686 cells, similar to FaDu cells, upregulated MBNL1 and impaired angiogenesis (Supplementary Fig. S5A − D). The pro-oncogenic role of human miR-130b-3p was further evaluated using a xenograft tumor model. As depicted in Fig. 6L − N, miR-130b-3p suppression markedly retarded tumor progression. IHC analysis of CD31 in the xenograft tumors confirmed that miR-130b-3p is a positive regulator of angiogenesis in vivo (Fig. 6O). Taken together, these data revealed that the mTORC1/STAT3/miR-130b-3p/MBNL1 signaling cascade is also present in human cancer cells.

### Inhibition of miR-130b-3p suppressed the growth of HNSCC PDX models

**Fig. 7:**
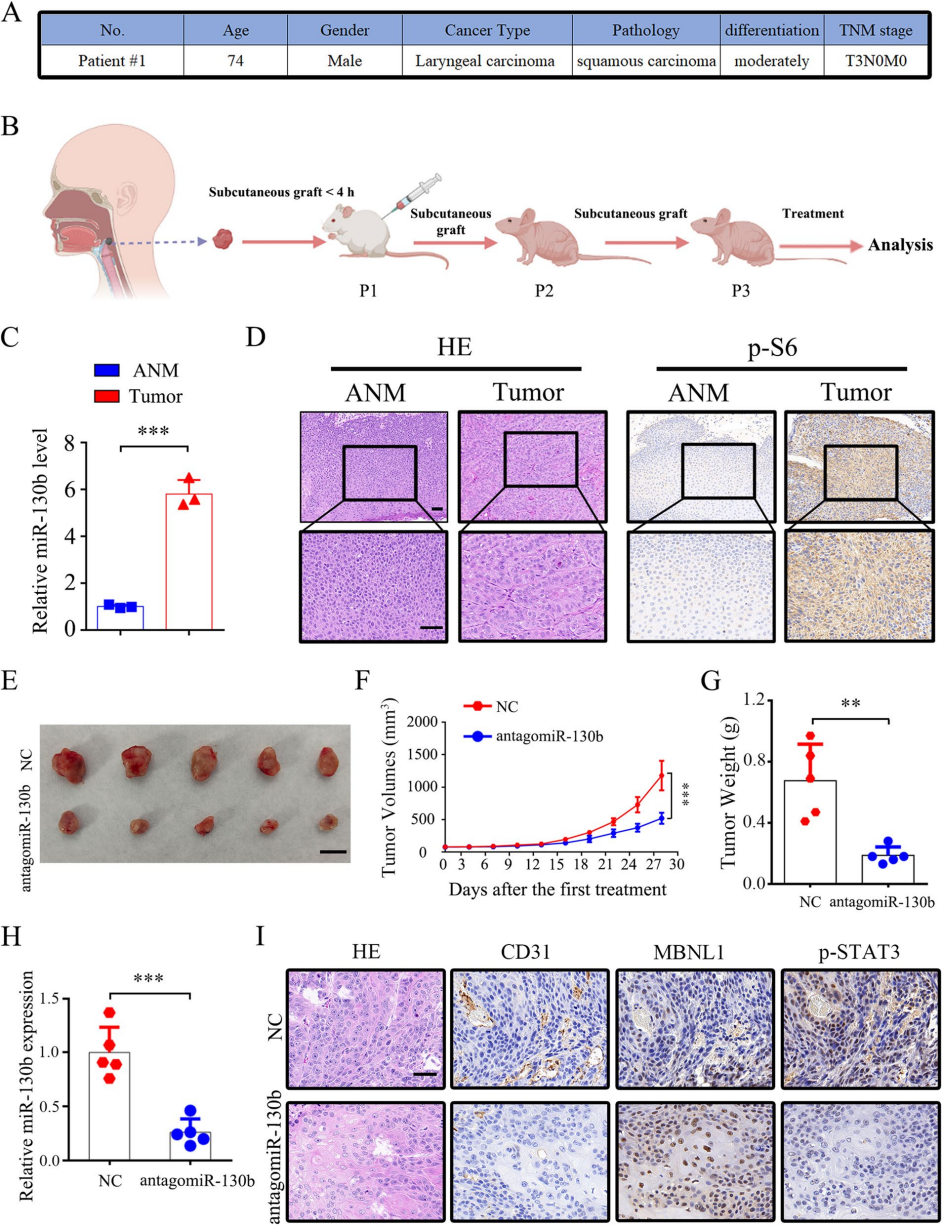
Effect of inhibition of miR-130b-3p on the growth of HNSCC PDX models. **A** Clinical characteristics of donor patients. **B** Graphic illustration of the generation of HNSCC PDX mouse models. **C** miR-130b-3p levels in the donor HNSCC tissues and paired ANM tissues were measured by qRT-PCR. **D** Representative IHC images of p-S6 staining from ANM or tumor tissues of donor patients. Scale bar, 50 μm. **E–G** PDX model tumors (*n* = 5 mice/group) treated with antagomiR-130b-3p (10 nmol/50 μL) or antagomiR-NC. **E** Tumor images. Scale bar, 1 cm. **F** Tumor volume. **G** Tumor weight. **H** miR-130-3p levels in the indicated tumor tissues were determined by qRT-PCR. **I** Representative HE and IHC staining for CD31, MBNL1, and p-STAT3 in the indicated PDX model tumors. Scale bar, 50 μm. Data indicate mean ± SD of 3–5 replicates. ** *P* < 0.01; *** *P* < 0.001

PDX tumor models can highly preserve the histological characteristics and heterogeneity of the original tumors [35, 36]. Therefore, to further validate the role of miR-130b-3p in the tumorigenesis of human cancers, a PDX model of HNSCC was constructed. As indicated in Fig. 7A − D, one fresh HNSCC tumor with activated mTORC1 and a high level of miR-130b-3p was chosen to establish the PDX model. The PDX model mice were intratumorally injected with antagomiR-130b-3p or antagomiR-NC once every 3 days. As indicated in Fig. 7E − G, antagomiR-130b-3p significantly delayed the growth of PDX tumors. The qRT-PCR analysis confirmed that miR-130b-3p was substantially decreased in the antagomiR-130b-3p-treated groups than in the antagomiR-NC-treated groups (Fig. 7H). Furthermore, the IHC staining of xenograft tissues indicated a marked increase in MBNL1 expression and a reduction in CD31 and p-STAT3 in the antagomiR-130b-3p-treated group as compared with the control group (Fig. 7I). Taken together, these results confirmed that miR-130b-3p inhibition could effectively inhibit the growth of HNSCC PDX models.

### MBNL1 inhibits the STAT3 activation

**Fig. 8:**
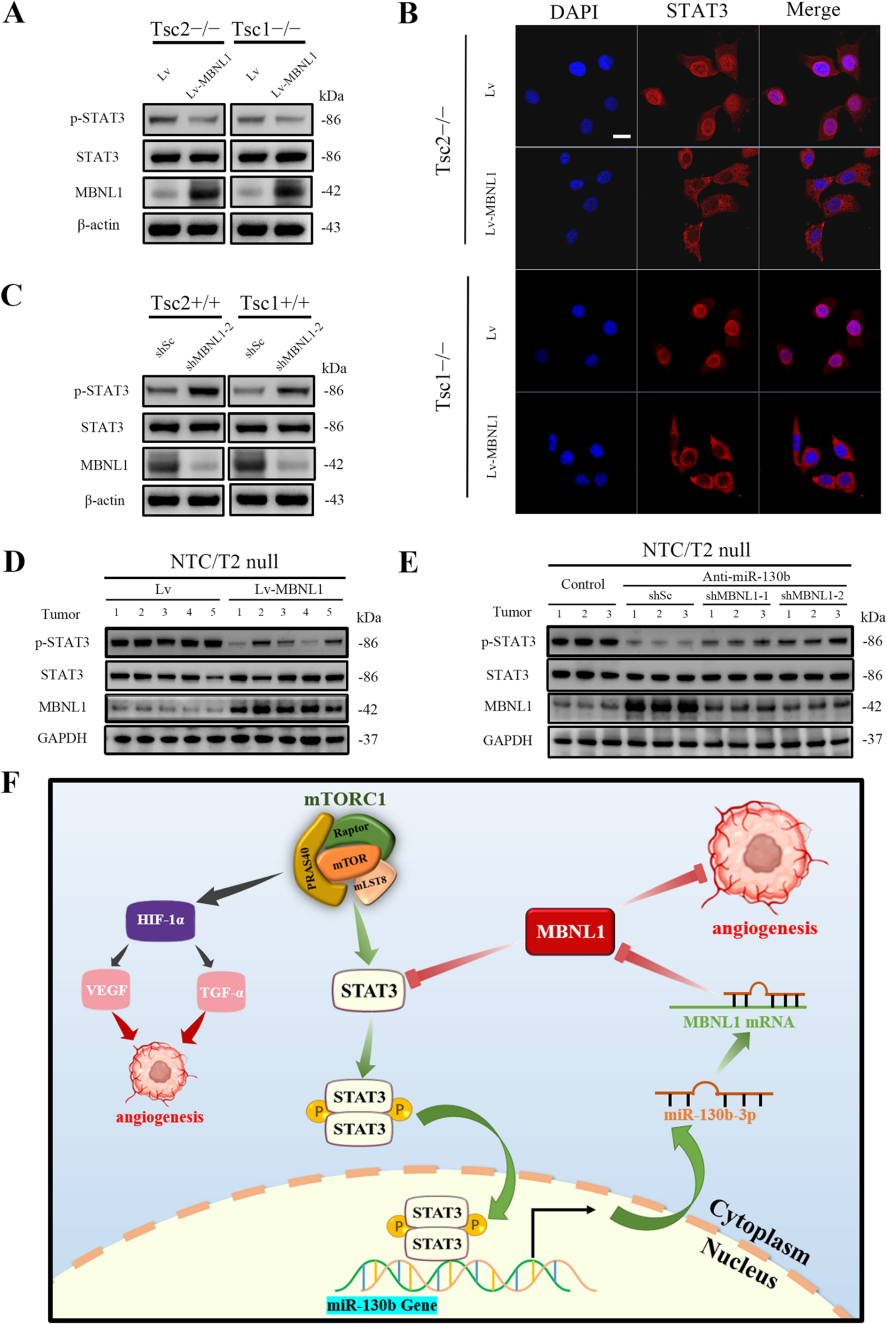
MBNL1 negatively regulates STAT3 activity. **A** and **B** Tsc2 − / − and Tsc1 − / − MEFs were transduced with a lentiviral vector expressing MBNL1 (Lv-MBNL1) or empty vector alone (Lv). **C** Tsc2 + / + and Tsc1 + / + MEFs were infected with lentiviruses expressing shRNAs targeting MBNL1 (shMBNL1-2) or a non-targeting shRNA (shSc). **A** and **C** Cell lysates were subjected to immunoblotting with the indicated antibodies. **B** The expression of STAT3 in the indicated cells was analyzed by an immunofluorescence assay. Scale bar, 20 μm. **D** and **E** Xenograft tumor tissues derived from the indicated cells were subjected to immunoblotting. **F** Schematic illustration of the STAT3/miR-130b-3p/MBNL1 feedback loop downstream of mTORC1 signaling promotes tumor angiogenesis

Next, we investigated whether decreased MBNL1 contributes to the enhancement of STAT3 activity caused by hyperactivation of mTORC1 signaling was investigated. As indicted in Fig. 8A, MBNL1 overexpression markedly attenuated STAT3 activity in Tsc2 − / − or Tsc1 − / − MEFs. Immunofluorescence analysis also confirmed that the nuclear level of STAT3 was dramatically reduced in response to MBNL1 overexpression (Fig. 8B). In contrast, knockdown of MBNL1 led to STAT3 activation in both Tsc2 + / + and Tsc1 + / + MEFs (Fig. 8C). Decreased p-STAT3 expression was observed in tumor tissues derived from MBNL1-overexpressing NTC/T2 null cells compared with the control cells (Fig. 8D). Moreover, decreased p-STAT3 expression in tumor tissues derived from anti-miR-130b-3p-expressing NTC/T2 null was partially rescued by knockdown of MBNL1 (Fig. 8E). Taken together, these data revealed that MBNL1 is a negative regulator of STAT3 and that miR-130b-3p-mediated downregulation of MBNL1 is, at least partially, responsible for the STAT3 activation in mTORC1-activated cells.

## Discussion

Hyperactivated mTORC1 promotes tumor angiogenesis, but its underlying mechanisms remain partially understood. In this study, mainly on the basis of Tsc2- or Tsc1-null MEFs, HNSCC cell lines, and HNSCC tissues, we identified miR-130b-3p as a novel downstream target of mTORC1. The positive correlation between mTORC1 and miR-130b-3p was also observed in multiple human cancer tissues. In addition, we illustrated that elevated miR-130b-3p contributed to angiogenesis and that tumor growth is driven by mTORC1. The vital role of miR-130b-3p in tumor angiogenesis has been illustrated recently in some human cancers. For example, Liao reported that miR-130b-3p was significantly upregulated and promoted angiogenesis in hepatocellular carcinoma [37]. Yan et al*.* demonstrated that exosomal miR-130b-3p derived from oral squamous cell carcinoma cells enhanced angiogenesis and tumor growth [38]. Our findings not only showed consistent results with these previous studies that miR-130b-3p is critical for angiogenesis but also revealed a new molecular link between mTORC1 activation and tumor angiogenesis. Because mTORC1 is frequently activated in hepatocellular carcinoma and oral squamous cell carcinoma owing to mutations in the RTK/PI3K/AKT pathway [39, 40], it may be that deregulated mTORC1 signaling in these cancers promotes miR-130b-3p expression and subsequent acceleration of angiogenesis and tumor progression. Therefore, miR-130b-3p is a potential therapeutic target for some mTORC1-related cancers.

In addition to angiogenesis, miR-130b-3p has been demonstrated to take part in various cellular processes involved in cancer progression, including cell proliferation, cell migration, apoptosis, and drug resistance [27, 41, 42]. Therefore, it is critical to examine how miR-130b-3p expression is controlled. Various transcription factors have been demonstrated to regulate the miR-130b gene expression. For example, Cannistraci and colleagues reported that c-Met activation increases miR-130b levels, which then promote prostate cancer metastasis and resistance to hormone ablation therapy [43]. Tong et al. demonstrated that metadherin, acting as a coactivator of NF-κB, promotes epithelial-mesenchymal transition (EMT) -like change and invasion of glioma cells through the upregulation of miR-130b transcription [44]. In addition, other transcription factors, such as TAp63, NF-YC, FOXM1, and p53, have also been reported to be involved in the transcriptional regulation of miR-130b in virous types of cells [45–48]. Here, we illustrated that STAT3, a well-known downstream effector of mTORC1, upregulates miR-130b-3p at the transcriptional level by directly binding the promoter of the miR-130b gene. We not only determined a novel transcription factor of miR-130b but also revealed that the upregulation of miR-130b-3p represents a new mechanism of angiogenesis driven by the over-activated mTORC1/STAT3 signaling pathway. In addition to transcriptional regulation, emerging evidence recently indicated that some lncRNAs and circular RNAs, such as H19 lncRNA and circSLC8A1, are also involved in the post-transcriptional regulation of miR-130b-3p [49, 50]. In the future, it will be interesting to investigate whether these noncoding RNAs are also involved in mTORC1-mediated regulation of miR-130b-3p.

MiRNAs are evolutionarily conserved small noncoding RNAs and are involved in tumorigenesis by targeting mRNAs for cleavage or translational repression [51, 52]. MiR-130b-3p has been determined to target multiple tumor suppressors, such as PTEN, HOXA5, and SASH1, in various types of cancer, resulting in cancer progression and treatment resistance [37, 38, 53]. In the current study, through the integration of bioinformatics and experimental strategies, we found that MBNL1 is an unreported target of miR-130b-3p. Furthermore, we confirmed that elevated miR-130b-3p driven by aberrantly activated mTORC1 signaling promotes angiogenesis and tumor growth through downregulation of MBNL1. The splicing regulator MBNL1 is a ubiquitously expressed RNA-binding protein [54]. MBNL1 was found to be downregulated in various common cancers, such as breast, lung, and stomach adenocarcinomas, and downregulation of MBNL1 predicted poor overall survival in patients with these cancers [55]. Although the specific role of MBNL1 in tumor angiogenesis has not been elucidated yet, several MBNL1-regulated genes are involved in angiogenesis [56]. Our findings not only confirmed that MBNL1 is a novel anti-angiogenic factor but also explained the regulatory molecular mechanism of MBNL1 expression. We propose that elevated miR-130b-3p resulting from hyperactivated mTORC1 signaling inhibits MBNL1 expression and then facilitates tumor angiogenesis and progression in some mTORC1-related cancers.

STAT3 is constitutively activated in various of cancer types and plays a vital role in tumor angiogenesis and expansion [57]. It is widely perceived that STAT3 acts as a key regulator of angiogenesis downstream of mTORC1 signaling [20, 58]. For example, Yang and colleagues demonstrated that blocking the mTORC1/STAT3 signaling pathway suppresses tumor angiogenesis [29]. Our previous study revealed that STAT3-mediated upregulation of brain expressed X-linked 2 is critical for angiogenesis induced by hyperactivated mTORC1 [19]. In addition, we have proposed that mTORC1 promotes glucose metabolism and suppresses cell differentiation via STAT3 activation [20, 21, 59]. However, the underlying mechanisms by which mTORC1 activates STAT3 are less elucidated. In the current study, we found that activated mTORC1 decreased MBNL1 expression through STAT3/miR-130b-3p pathway activation. Interestingly, ectopic expression of MBNL1 attenuates the phosphorylation and activation of STAT3 in Tsc1- or Tsc2-deficient cells, whereas MBNL1 depletion facilitates STAT3 activation. Thus, our study illustrated a feedback loop between STAT3 and MBNL1 downstream of mTORC1 signaling and provided the first evidence that mTORC1 activates STAT3, at least in part, by MBNL1 inhibition. Together with our previous findings that mTORC1 upregulates STAT3 protein levels through miR-125b-5p suppression and mTORC1 activates STAT3 under the promotion of EGFR expression [25, 32], our results open a possibility that multiple molecular mechanisms are involved in mTORC1-dependent regulation of STAT3. However, it remains to be verified how MBNL1 regulates the activity of STAT3. A previous study has demonstrated that knockdown of MBNL1 led to c-Jun N-terminal kinase (JNK) activation [55]. Because JNK is a well-known positive regulator of STAT3 [60, 61], decreased MBNL1 may contribute to the enhancement of STAT3 activity via JNK activation in mTORC1-activated cells. Future work, however, is required to examine this possibility.

## Conclusions

We demonstrate that aberrantly activated mTORC1 contributes to angiogenesis and tumor growth by regulating of the STAT3/miR-130b-3p/MBNL1 feedback loop (Fig. 8F). Our findings help in the elucidation of the molecular mechanism by which dysregulated mTORC1 signaling drives tumor angiogenesis, indicating that the components in the STAT3/miR-130b-3p/MBNL1 loop signaling pathway may be targeted for the treatment of mTORC1-related tumors.

## Supplementary Information

**Additional file 1: Fig. S1.** Inhibition of miR-130b-3p suppresses angiogenesis. **Fig. S2.** Overexpression of MBNL1 inhibits angiogenesis. **Fig. S3.** miR-130b-3p promotes angiogenesis through downregulation of MBNL1. **Fig. S4.** miR-130b-3p was upregulated and positively correlated with mTORC1 signaling in multiple human cancers. **Fig. S5.** Inhibition of mTORC1/miR-130b-3p axis impairs angiogenesis.

**Additional file 2: Supplementary Table 1.** Clinical features of 60 HNSCC patients. **Supplementary Table 2.** Oligonucleotide sequences used in this study. **Supplementary Table 3.** Antibodies used in this study. **Supplementary Table 4.** Primer sequences used for qRT-PCR in this study. **Supplementary Table 5.** Primers used for luciferase reporter in this study. **Supplementary Table 6.** Primers used for ChIP Assays in this study.

**Additional file 3: Supplementary Table S7.** List of the predict target genes of miR-130b-3p from prediction softwares TargetScan, RNA22, PicTar, miRDB and miRWalk.

**Additional file 4: Supplementary Table S8.** List of the downregulated differentially expressed genes from RNA sequencing results in Tsc2-/- MEFs and Tsc2+/+ MEFs. All genes were identified with FDR-adjusted *p*-value<0.05 and absolute value of log2(FC)>1.

**Additional file 5: Supplementary Table 9.** List of the downregulated differentially expressed genes from DEGs in angiofibroma(AF) of TSC patients vs. normal skin. All genes were identified with FDR-adjusted *p*-value < 0.05 and absolute value of log2(FC) > 1.

## Data Availability

The data supporting the conclusions of this article have been given in this article and its additional files.

## Abbreviations

mTOR: Mammalian target of rapamycin
mTORC1: Mammalian target of rapamycin complex 1
mTORC2: Mammalian target of rapamycin complex 2
TSC1: Heterodimer tuberous sclerosis 1
TSC2: Heterodimer tuberous sclerosis 2
miR-130b-3p: Micro-RNA-130b-3p
PDX: Patient-derived xenograft
STAT3: Activator of transcription 3
MBNL1: Muscleblind-like protein 1
HNSCC: Head and neck squamous cell carcinoma
NOK: Normal oral keratinocyte
HUVECs: Human umbilical vein endothelial cells. Rheb: Ras homologue enriched in brain
GAP: GTPase activating
ANM: Adjacent normal mucosal
MEFs: Mouse embryonic fibroblasts
DEGs: Differentially expressed genes
3´-UTR: 3´Untranslated region 3´-UTR
MOI: Multiplicity of infection
CM: Cell-derived conditioned medium
CAM: Chicken chorioallantoic membrane
TCGA: The Cancer Genome Atlas
AGO2: Argonaut proteins 2
qRT-PCR: Quantitative real-time PCR
ChIP: Chromatin immunoprecipitation
ELISA: Enzyme-linked immunosorbent assay
RIP: RNA immunoprecipitation
IHC: Immunohistochemistry
GSEA: Gene Set Enrichment Analysis
AF: Angiofibroma
DMSO: Dimethyl sulfoxide
DMEM: Dulbecco's Modified Eagle's Medium
