# Supplementary material for: STAT3/miR-130b-3p/MBNL1 feedback loop regulated by mTORC1 signaling promotes angiogenesis and tumor growth
Source: J Exp Clin Cancer Res. 2022 Oct 11;41:297. doi: 10.1186/s13046-022-02513-z (PMC9552455; doi:10.1186/s13046-022-02513-z)
Supplement: Supplementary file 1 — Additional file 1: Fig. S1. Inhibition of miR-130b-3p suppresses angiogenesis. Fig. S2. Overexpression of MBNL1 inhibits angiogenesis. Fig. S3. miR-130b-3p promotes angiogenesis through downregulation of MBNL1. Fig. S4. miR-130b-3p was upregulated and positively correlated with mTORC1 signaling in multiple human cancers. Fig. S5. Inhibition of mTORC1/miR-130b-3p axis impairs angiogenesis. [file 13046_2022_2513_MOESM1_ESM.docx]

**Supplementary Figures**

**
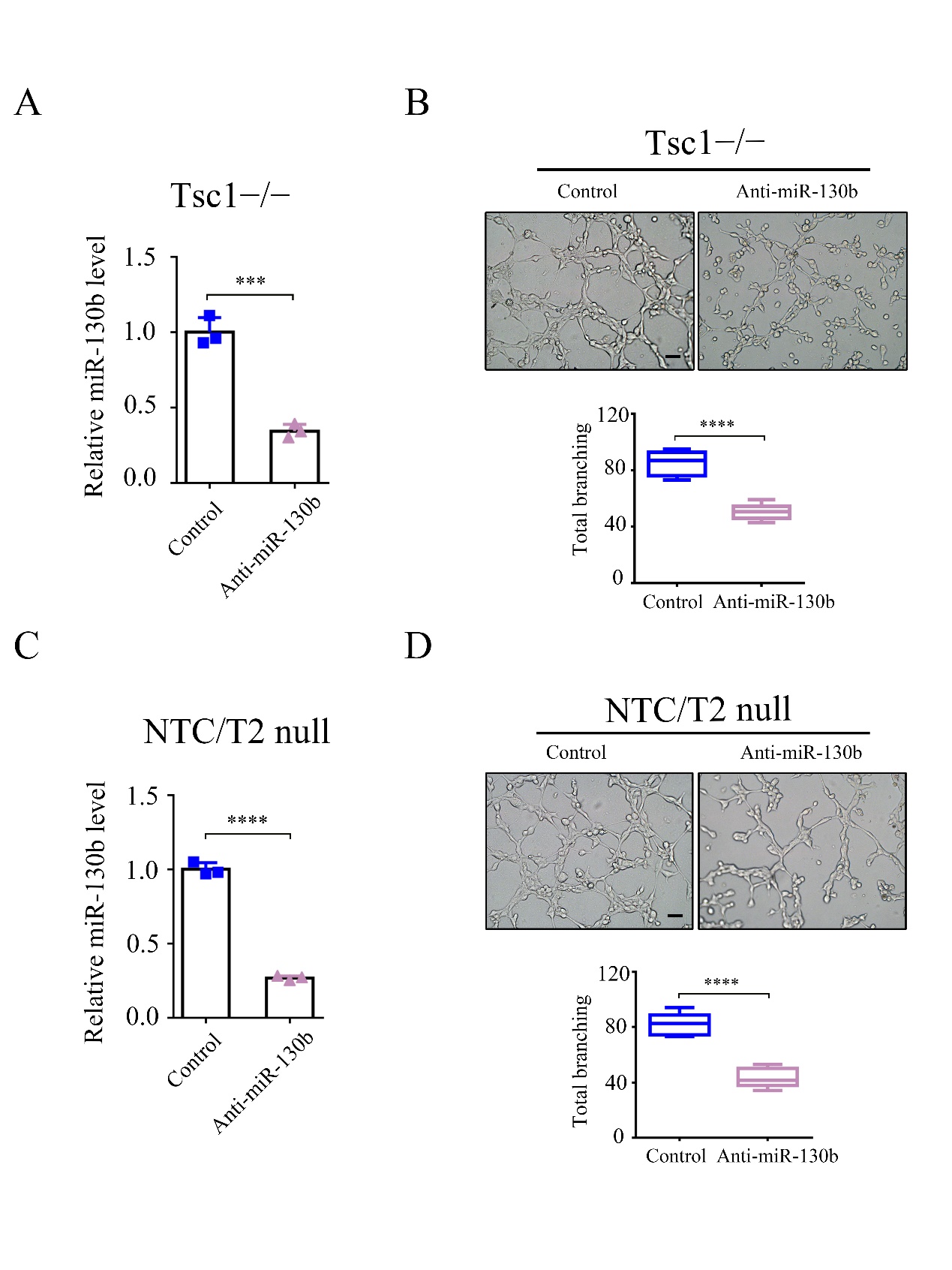
Supplementary Fig. S1: Inhibition of miR-130b-3p suppresses angiogenesis.**

Tsc1−/− MEFs or NTC/T2 null cells were transduced with lentiviruses expressing anti-miR-130b-3p or a control vector. (A and C) The expression of miR-130-3p was determined by qRT-PCR. (B and D) The impact of anti-miR-130b-3p on tube formation ability was detected by tube formation assay. Representative images (up panels) and quantifications (low panels) are shown. Scale bar, 50 μm. Data indicate mean ± SD of 3-5 replicates. *** *P*<0.001; **** *P*<0.0001.

**
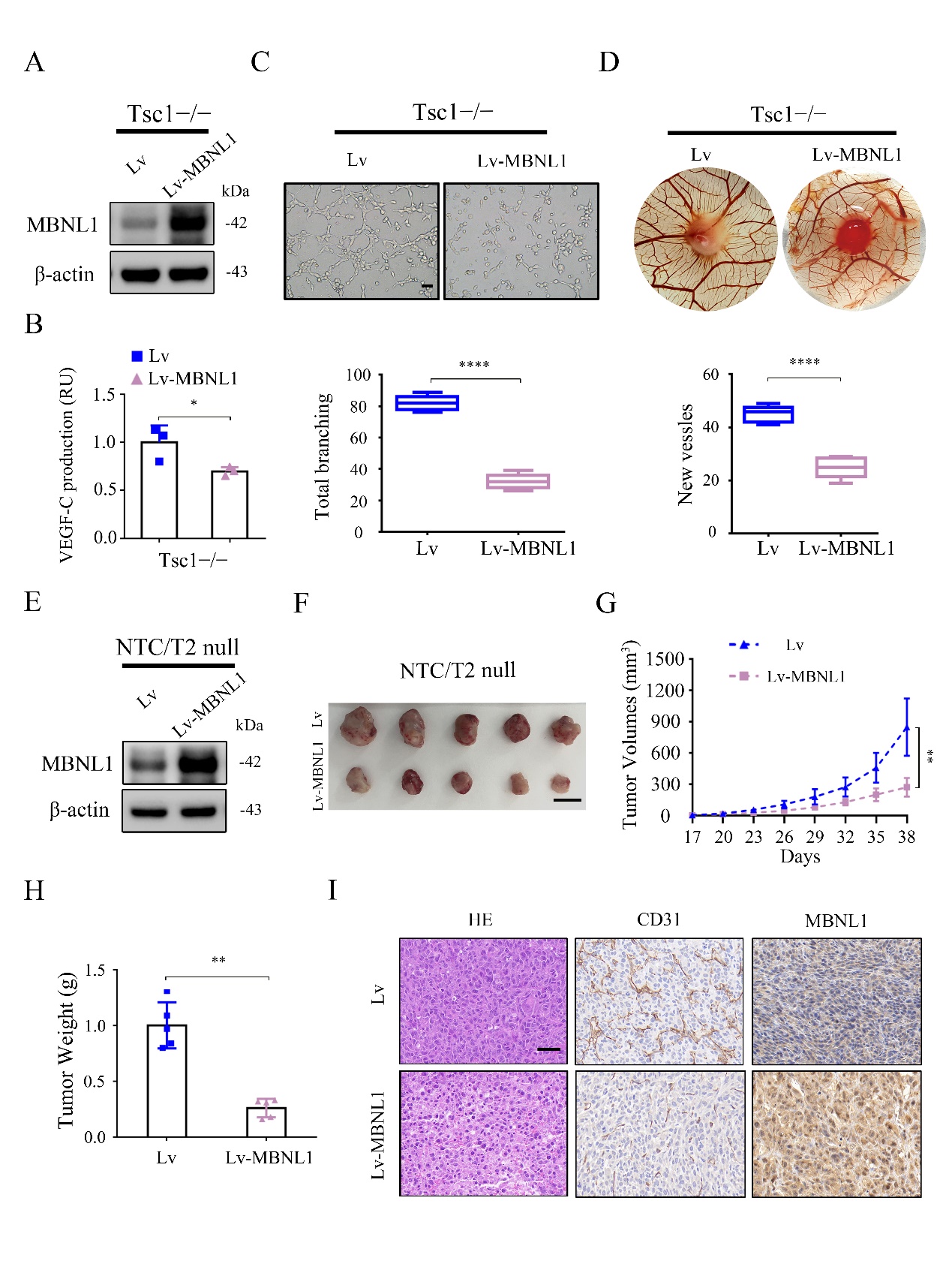
Supplementary Fig. S2: Overexpression of MBNL1 inhibits angiogenesis.**

(A-D) Tsc1−/− MEFs were infected with lentiviruses carrying either MBNL1 (Lv-MBNL1) or an empty vector control (Lv). (A) The expression of MBNL1 was determined by western blot. (B) Cell culture supernatants were subjected to ELISA for VEGF-C expression. (C and D) The effect on angiogenesis was detected by tube formation assay (C, scale bar, 50 μm) and CAM assay (D). Representative images (up panels) and quantifications (low panels) are shown. (E) Lv-MBNL1- or Lv-expressing NTC/T2 null cells were subjected to immunoblotting. (F-I) Xenograft tumor growth of mice harboring NTC/T2 null cells with Lv-MBNL1 or the control Lv. (F) Tumor pictures. Scale bar, 1cm. (G) Tumor growth curves. (H) Tumor weight. (I) Representative HE and IHC staining for CD31 and MBNL1 in the indicated tumor tissues. Scale bar, 50 μm. Data indicate mean ± SD of 3-5 replicates. * *P*<0.05; ** *P*<0.01; **** *P*<0.0001.

**
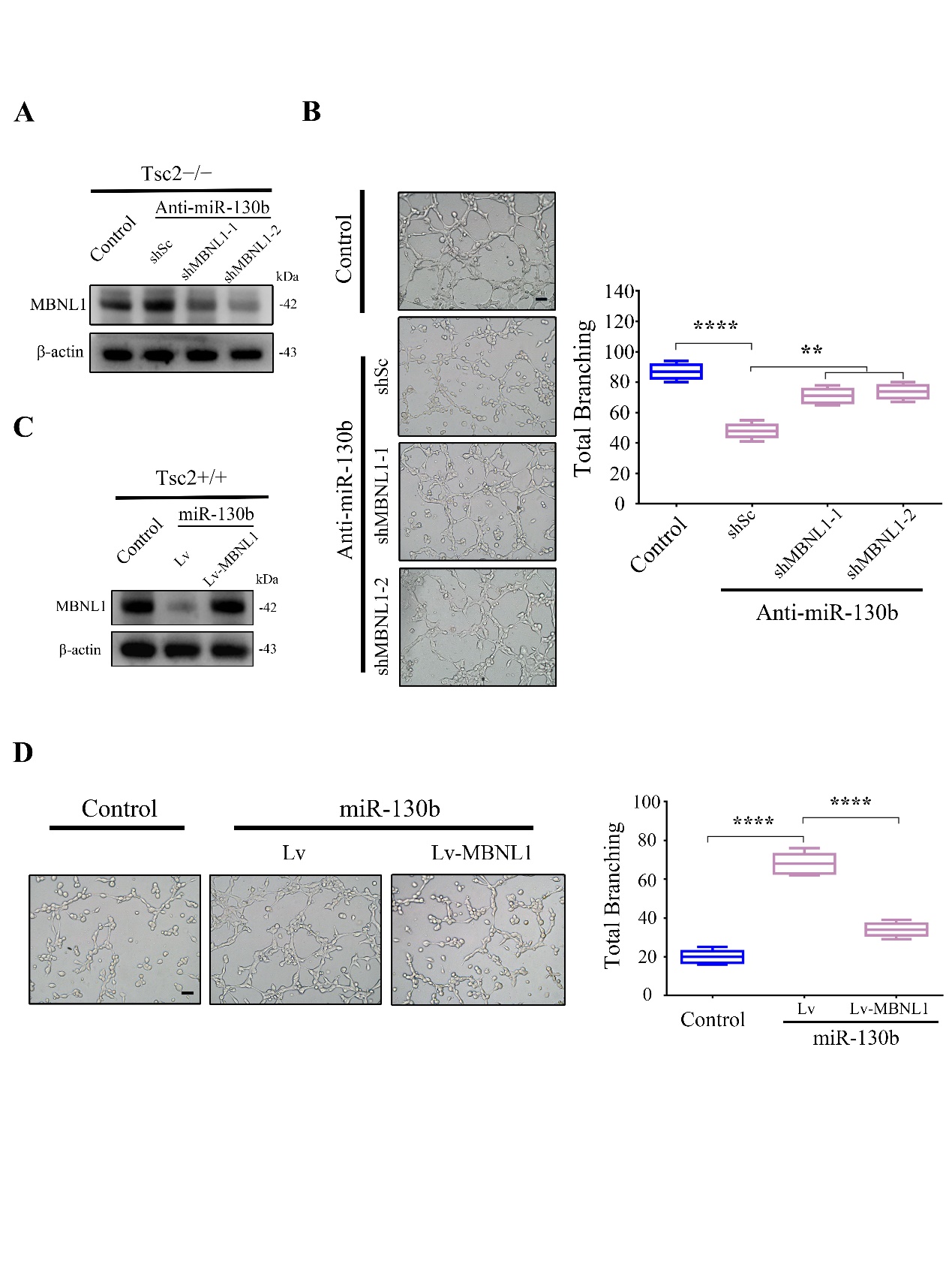
Supplementary Fig. S3: miR-130b-3p promotes angiogenesis through downregulation of MBNL1.**

(A and B) anti-miR-130b-3p-expressing Tsc2−/− MEFs were infected with lentivirus harboring MBNL1 shRNAs (shMBNL1-1 and shMBNL1-2) or a scrambled shRNA (shSc). (C and D) miR-130b-3p-expressing Tsc2+/+ MEFs were infected with lentiviruses carrying an empty vector (Lv) or expression vectors for MBNL1 (Lv-MBNL1). (A and C) Cell lysates were subjected to immunoblotting with the indicated antibodies. (B and D, scale bar, 50 μm) The impact on angiogenesis was detected by tube formation assay. Representative images (left panels) and quantifications (right panels) are shown. Data indicate mean ± SD of 3-5 replicates. ** *P*<0.01; **** *P*<0.0001.

**
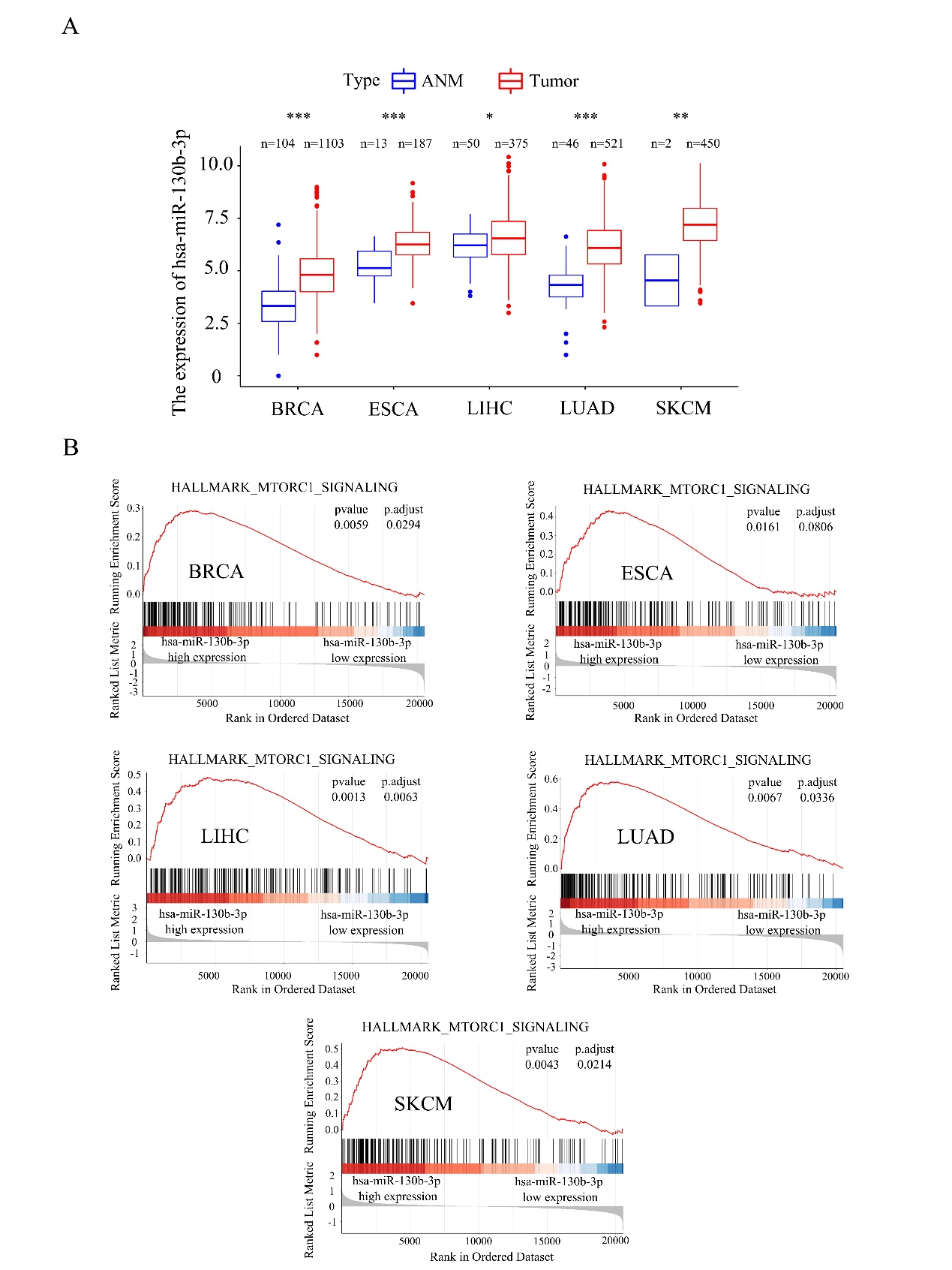
Supplementary Fig. S4: miR-130b-3p was upregulated and positively correlated with mTORC1 signaling in multiple human cancers.**

(A) miR-130b-3p levels were analyzed in BRCA, ESCA, LIHC, LUAD, SKCM and their corresponding normal tissues using TCGA datasets. (B) GSEA comparing the gene sets positively regulated by mTORC1 signaling in miR-130b-3p-high and miR-130b-3p-low BRCA, ESCA, LIHC, LUAD and SKCM patients, based on TCGA datasets. * *P*<0.05; ** *P*<0.01; *** *P*<0.001.

**
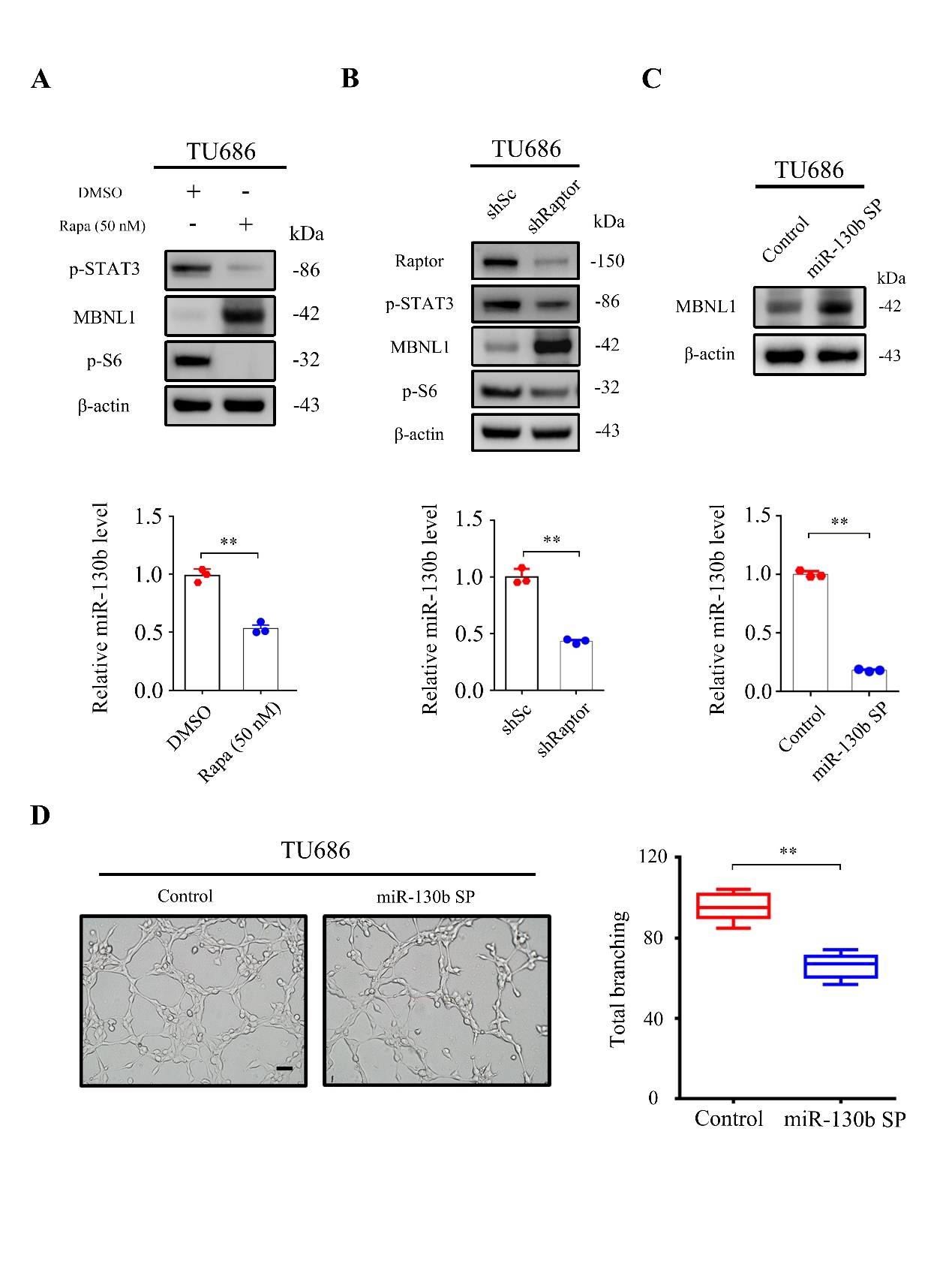
Supplementary Fig. S5: Inhibition of mTORC1/miR-130b-3p axis impairs angiogenesis.**

(A) TU686 cells were treated with 50 nM rapamycin for 24 hours. (B) TU686 cells were infected with lentivirus expressing shRNAs targeting Raptor (shRaptor) or a control shRNA (shSc). (C) TU686 cells were infected with lentiviruses expressing miRNA sponges targeting miR-130b-3p (miR-130b SP) or a control vector. (A-C) Cell lysates were subjected to immunoblotting with the indicated antibodies (up panels); the expression of miR-130b-3p was detected by qRT-PCR (low panels). (D) The effect of anti-miR-130b-3p on angiogenesis was tested by tube formation assay. Representative images (left panel) and quantifications (right panel) are shown. Scale bar, 50 μm. Data indicate mean ± SD of 3-5 replicates ** *P*<0.01; **** *P*<0.0001.
