# Supplementary material for: STAT3/miR-130b-3p/MBNL1 feedback loop regulated by mTORC1 signaling promotes angiogenesis and tumor growth
Source: J Exp Clin Cancer Res. 2022 Oct 11;41:297. doi: 10.1186/s13046-022-02513-z (PMC9552455; doi:10.1186/s13046-022-02513-z)
Supplement: Supplementary file 2 — Additional file 2: Supplementary Table 1. Clinical features of 60 HNSCC patients. Supplementary Table 2. Oligonucleotide sequences used in this study. Supplementary Table 3. Antibodies used in this study. Supplementary Table 4. Primer sequences used for qRT-PCR in this study. Supplementary Table 5. Primers used for luciferase reporter in this study. Supplementary Table 6. Primers used for ChIP Assays in this study. [file 13046_2022_2513_MOESM2_ESM.docx]

**Supplementary Table 1. Clinical features of 60 HNSCC patients.**

| **Parameters** | **Number of Cases(%)** |
| --- | --- |
| Age |  |
| < 60 | 22（36.7） |
| ≥ 60 | 38（63.3） |
| Gender |  |
| Male | 54（90.0） |
| FeMale | 6（10.0） |
| cancer type |  |
| laryngeal carcinoma | 34 (56.7) |
| hypopharyngeal carcinoma | 26 (43.3) |
| T Stage**^^[[1]](#footnote-1)^^** |  |
| T1 | 13（21.7） |
| T2 | 16（26.7） |
| T3 | 17（28.3） |
| T4 | 14（23.3） |
| lymph node metastasis |  |
| N0 | 45（75.0） |
| N+ | 15（25.0） |
| Distant metastasis |  |
| M0 | 58（96.7） |
| M1 | 2（ 3.3） |

| **Supplementary Table 2. Oligonucleotide sequences used in this study.** | |
| --- | --- |
| **Note** | **Sequences ( 5'-3' )** |
| (mus)/(human) shSc | AATCGCATAGCGTATGCCG |
| (mus) shRaptor | GGACAACGGTCACAAGTAC |
| (mus) shRictor | GCCCTACAGCCTTCATTTA |
| (mus) shSTAT3 | CTGGATAACTTCATTAGCA |
| (mus) shMBNL1-1 | GGCGGAAUAACUUGAUUCATT |
| (mus) shMBNL1-2 | GGGAAUUCCUCAAGCUGUATT |
| (human) shRaptor | GGACAACGGCCACAAGTAC |
| (mus)/(human) Control | TTCTCCGAACGTGTCACGT |
| (mus) Anti-miR-130b-3p | ATGCCCTTTCATCATTGCACTG |
| (human) miR-130b SP | GATCCATGCCCTTTCATCATTGCACTGCGATATGCCCTTTCATCATT GCACTGTCACATGCCCTTTCATCATTGCACTGTTTTTTGAATT |
| (mus) pre-miR-130b (82 bp) | GGCTTGTTGGACACTCTTTCCCTGTTGCACTACTGTGGGCCTCTGGGAAGCAGTGCAATGATGAAAGGGCATCTGTCGGGCC |

| **Supplementary Table 3. Antibodies used in this study.** | | | | |
| --- | --- | --- | --- | --- |
| **Antibody** | **Source** | | **Application** | |
| TSC2 | | Cell Signaling Technology (Cat #4308S) | | 1:1000 for WB |
| TSC1 | | Cell Signaling Technology (Cat #6935S) | | 1:1000 for WB |
| p-S6(Ser235/236) | | Cell Signaling Technology (Cat #4857S) | | 1:1000 for WB; 1:400 for IHC |
| β-actin | | Cell Signaling Technology (Cat #3700S) | | 1:1000 for WB |
| Rictor | | Cell Signaling Technology (Cat #2114S) | | 1:1000 for WB |
| Raptor | | Cell Signaling Technology (Cat #2280S) | | 1:1000 for WB |
| p-mTOR(Ser2448) | | Cell Signaling Technology (Cat #5536S) | | 1:1000 for WB |
| p-STAT3(Tyr705) | | Cell Signaling Technology (Cat #9145S) | | 1:1000 for WB; 1:100 for IHC  1:100 for CHIP |
| STAT3 | | Cell Signaling Technology (Cat #12640S) | | 1:1000 for WB; 1:400 for IHC;  1:800 for IF |
| MBNL1 | | Proteintech (Cat #66837-1-Ig) | | 1:1000 for WB; 1:500 for IHC |
| GAPDH | | Cell Signaling Technology (Cat #5174S) | | 1:1000 for WB |
| CD31 | | Abcam (Cat #ab182981) | | 1:500 for IHC |
| Ki67 | | Cell Signaling Technology (Cat #9027S) | | 1:500 for IHC |

| **Supplementary Table 4. Primer sequences used for qRT-PCR in this study.** | | |
| --- | --- | --- |
| **Primer name** | **Sequence (5' to 3')** | **Source** |
| (mus) miR-130b-3p forward | CAGTCCACCAGTGCAATGATG | GenePharma, Shanghai, China |
| (mus) miR-130b-3p reverse | TATGCTTGTTCTCGTCTCTGTGTC | GenePharma, Shanghai, China |
| (mus) miR-199a-5p forward | CATCTCTCTACTCCCAGTGTTCAG | GenePharma, Shanghai, China |
| (mus) miR-199a-5p reverse | TATGGTTGTTCTGCTCTCTGTGTC | GenePharma, Shanghai, China |
| (mus) U6 forward | CAGCACATATACTAAAATTGGAACG | GenePharma, Shanghai, China |
| (mus) U6 reverse | ACGAATTTGCGTGTCATCC | GenePharma, Shanghai, China |
| (mus) β-actin forward | AAATCGTGCGTGACATCAAA | GenePharma, Shanghai, China |
| (mus) β-actin reverse | AAGGAAGGCTGGAAAAGAGC | GenePharma, Shanghai, China |
| (mus) MBNL1 forward | CACTGAAAGGTCGTTGCTCCA | TsingKe，Hefei，China |
| (mus) MBNL1 reverse | CGCCCATTTATCTCTAACTGTGT | TsingKe，Hefei，China |
| (mus) MBNL1 forward  (For RIP) | TGAGAATTTTAGCGTGTGTG | TsingKe，Hefei，China |
| (mus) MBNL1 reverse  (For RIP) | TTGCTCAGTCTGTCATTTCATT | TsingKe，Hefei，China |
| (mus) MMP13 forward | TGTTTGCAGAGCACTACTTGAA | TsingKe，Hefei，China |
| (mus) MMP13 reverse | CAGTCACCTCTAAGCCAAAGAAA | TsingKe，Hefei，China |
| (mus) VEGFC forward | GAGGTCAAGGCTTTTGAAGGC | TsingKe，Hefei，China |
| (mus) VEGFC reverse | CTGTCCTGGTATTGAGGGTGG | TsingKe，Hefei，China |
| (mus) BCL2 forward | GCTACCGTCGTGACTTCGC | TsingKe，Hefei，China |
| (mus) BCL2 reverse | CCCCACCGAACTCAAAGAAGG | TsingKe，Hefei，China |
| (mus) IL18 forward | GTGAACCCCAGACCAGACTG | TsingKe，Hefei，China |
| (mus) IL18 reverse | CCTGGAACACGTTTCTGAAAGA | TsingKe，Hefei，China |
| (human) miR-130b-3p forward | CAGTCCACCAGTGCAATGATG | GenePharma, Shanghai, China |
| (human) miR-130b-3p reverse | TATGCTTGTTCTCGTCTCTGTGTC | GenePharma, Shanghai, China |
| (human) U6 forward | CAGCACATATACTAAAATTGGAACG | GenePharma, Shanghai, China |
| (human) U6 reverse | ACGAATTTGCGTGTCATCC | GenePharma, Shanghai, China |
| (human) GAPDH forward | GGAAGGTGAAGGTCGGAGTC | TsingKe，Hefei，China |
| (human) GAPDH reverse | TCGCCCCACTTGATTTTGGA | TsingKe，Hefei，China |

| **Supplementary Table 5. Primers used for luciferase reporter in this study.** | | |
| --- | --- | --- |
| **Gene** | **Wild type** | |
|  | **Forward primers (5'-3')** | **Reverse primers (5'-3')** |
| miR-130b | GGGGTACCCGAGGGAG AGAGGGCGCCACGAG | GAAGATCTGCACGTG AGGCCCTTGGAGTCCG |
| MBNL1 | CTAGCTAGCGCTGCATAATCA ATAAATAGAACAAGGGAC | GCTCTAGAGCGACGTG TTTGAAGGGTATGGAGT |
| **Gene** | **Mutant Type** | |
|  | **Forward primers (5'-3')** | **Reverse primers (5'-3')** |
| miR-130b | CTCCAGATGCGCCCAA GCCCTAGTTCTGCTCTTC | TTTCCCTGGCCTCACCGC |
| MBNL1 | TGATCTGTATATAGT CAAAAGAGAG | CGTTAAAGTCAACCTTCTCTCTAG |

| **Supplementary Table 6. Primers used for ChIP Assays in this study.** | | |
| --- | --- | --- |
|  | **Sense (5'-3')** | **Antisense (5'-3')** |
| Site #1  (PCR) | 5'-GCCGCCGCCTACCTGGATCT-3' | 5'-TCCGGTCCTTGGTCCATTCCC-3' |
| Site #2  (PCR) | 5'-GGTCAGAAGAGGGTGTTGGATTT-3' | 5'-TGAGTTTGGGGGCTGGAGAG-3' |
| NBR  (qRT-PCR) | 5'-GAACTTCTGATCCTCCTGCCTCTAT- 3' | 5'-CCCACAAAGTAGCCAAACGTCT- 3' |
| PBR  (qRT-PCR) | 5'-CACGTGAGTAACTGGTCTGGGATAGG-3' | 5'-CCGGTCCTTGGTCCATTCCC-3' |

1. TNM Staging is referring to the AJCC 8th edition TNM Staging Criteria. [↑](#footnote-ref-1)
